# From biomechanics to pathology: predicting axonal injury from patterns of strain after traumatic brain injury

**DOI:** 10.1093/brain/awaa336

**Published:** 2021-01-17

**Authors:** Cornelius K Donat, Maria Yanez Lopez, Magdalena Sastre, Nicoleta Baxan, Marc Goldfinger, Reneira Seeamber, Franziska Müller, Polly Davies, Peter Hellyer, Petros Siegkas, Steve Gentleman, David J Sharp, Mazdak Ghajari

**Affiliations:** 1 Department of Brain Sciences, Faculty of Medicine, Imperial College London, London, UK; 2 Royal British Legion Centre for Blast Injury Studies, Imperial College London, London, UK; 3 Centre for the Developing Brain, School of Biomedical Engineering and Imaging Sciences, King’s College London, London, UK; 4 Biological Imaging Centre, Central Biomedical Services, Imperial College London, London, UK; 5 Centre for Neuroimaging Sciences, King’s College London, London, UK; 6 Design Engineering, Imperial College London, UK; 7 UK Dementia Research Institute, Care Research and Technology Centre; Imperial College London, London, UK

**Keywords:** finite element modelling, traumatic brain injury, controlled cortical impact, diffusion tensor imaging, quantitative histology

## Abstract

The relationship between biomechanical forces and neuropathology is key to understanding traumatic brain injury. White matter tracts are damaged by high shear forces during impact, resulting in axonal injury, a key determinant of long-term clinical outcomes. However, the relationship between biomechanical forces and patterns of white matter injuries, associated with persistent diffusion MRI abnormalities, is poorly understood. This limits the ability to predict the severity of head injuries and the design of appropriate protection. Our previously developed human finite element model of head injury predicted the location of post-traumatic neurodegeneration. A similar rat model now allows us to experimentally test whether strain patterns calculated by the model predicts *in vivo* MRI and histology changes. Using a controlled cortical impact, mild and moderate injuries (1 and 2 mm) were performed. Focal and axonal injuries were quantified with volumetric and diffusion 9.4 T MRI at 2 weeks post injury. Detailed analysis of the corpus callosum was conducted using multi-shell diffusion MRI and histopathology. Microglia and astrocyte density, including process parameters, along with white matter structural integrity and neurofilament expression were determined by quantitative immunohistochemistry. Linear mixed effects regression analyses for strain and strain rate with the employed outcome measures were used to ascertain how well immediate biomechanics could explain MRI and histology changes. The spatial pattern of mechanical strain and strain rate in the injured cortex shows good agreement with the probability maps of focal lesions derived from volumetric MRI. Diffusion metrics showed abnormalities in the corpus callosum, indicating white matter changes in the segments subjected to high strain, as predicted by the model. The same segments also exhibited a severity-dependent increase in glia cell density, white matter thinning and reduced neurofilament expression. Linear mixed effects regression analyses showed that mechanical strain and strain rate were significant predictors of *in vivo* MRI and histology changes. Specifically, strain and strain rate respectively explained 33% and 28% of the reduction in fractional anisotropy, 51% and 29% of the change in neurofilament expression and 51% and 30% of microglia density changes. The work provides evidence that strain and strain rate in the first milliseconds after injury are important factors in determining patterns of glial and axonal injury and serve as experimental validators of our computational model of traumatic brain injury. Our results provide support for the use of this model in understanding the relationship of biomechanics and neuropathology and can guide the development of head protection systems, such as airbags and helmets.

## Teaser

The relationship between biomechanics and pathology after brain injury is poorly understood. Using computational modelling, Donat *et al.* show that mechanical strain immediately after injury predicts the extent of post-traumatic axonal pathology, helping us better understand outcomes following head impacts in humans.

## Introduction

Traumatic brain injury (TBI) involves the rapid transfer of mechanical forces onto the head and brain. This loading results in immediate deformations, causing axonal disconnection, neuronal loss, vascular damage, and release of excitatory neurotransmitters. Secondary injuries follow, caused by delayed molecular cascades, aggravating axonal damage and neurodegeneration (Prins *et al.*, 2013). Diffuse axonal injury is common after head injury and leads to persistent neurological and psychiatric disability (Scheid *et al.*, 2006; Moen *et al.*, 2012; Sharp *et al.*, 2014; Hill *et al.*, 2016). Initial loading conditions following head impacts are assumed to determine the location and extent of focal and diffuse axonal injury, but there is little understanding of the threshold for biomechanical forces over which damage to the brain is produced.

Understanding the relationship between patterns of biomechanical force and the location of axonal injury is key to predicting the effects of different types of head injury, as well as designing brain protection systems—such as airbags and helmets—that are optimized for the prevention of axonal injury. White matter tracts are particularly vulnerable to mechanical loading via different mechanisms (Hill *et al.*, 2016). If exposed to loading above a certain threshold, primary axotomy and damage will result from shear and stretch (Smith *et al.*, 2003; Di Pietro *et al.*, 2013), even though this is more prominent in contusions and lacerations (Christman *et al.*, 1994). Neurofilaments are a family of proteins abundantly expressed in the axons of the white matter, such as the corpus callosum, where they form a major constituent of the cytoskeleton (Lépinoux-Chambaud and Eyer, 2013). Consequently, axonal damage and disruption after TBI will result in degradation of neurofilaments and clearing into the CSF and blood, where their presence can be detected in animal models and patients (Posmantur *et al.*, 1994; Iverson *et al.*, 2019; Yang *et al.*, 2019; Dickstein *et al.*, 2020). This makes neurofilaments, especially when derived from CSF and blood, a promising biomarker (Zetterberg *et al.*, 2013; Hiskens *et al.*, 2020). Once axonal damage occurs, it can subsequently result in excitotoxicity, cytoskeletal degradation and release of damage associated molecular patterns (DAMPs), causing secondary axotomy or axonal degeneration (Buki and Povlishock, 2006; Braun *et al.*, 2017). This usually elicits a profound immune response, e.g. from glia cells. Areas of axonal injury are often associated with a microglial and astrocytic response in both animal models and patients, still detectable years after injury (Csuka *et al.*, 2000; Wang *et al.*, 2013*a*; Lafrenaye *et al.*, 2015; Scott *et al.*, 2015). However, the exact nature of this response, either immediately after injury or long-term, remains to be elucidated and could be protective, neutral or deleterious, depending on the spatio-temporal context.

Furthermore, axonal properties such as orientation and location, myelination and length predict the likelihood of axonal injury, with unmyelinated long axons being more vulnerable (Reeves *et al.*, 2005; Staal and Vickers, 2011; Marion *et al.*, 2018). Differences in the mechanical properties of white/grey matter and CSF compartments also influence injury, with forces concentrated at tissue interfaces (Drew and Drew, 2004; Cloots *et al.*, 2013; Yu *et al*., 2020).

Computational models of TBI provide predictions of the forces within the first milliseconds after the impact. The finite element (FE) method allows prediction of strains and strain rates with high spatiotemporal resolution (Chu *et al.*, 1994; Zhang *et al.*, 2001; Kleiven and Hardy, 2002; Shuaeib *et al.*, 2002; Willinger and Baumgartner, 2010; Ghajari *et al.*, 2017). We have recently developed a high-fidelity model of the human brain with gyral anatomy and a range of tissue types. This allows detailed prediction of biomechanical forces seen in different tissues after head injury (Ghajari *et al.*, 2017). Our modelling showed that various types of TBI result in high strains and strain rates concentrated in the depths of the sulci, the location associated with chronic traumatic encephalopathy. This suggests that computational modelling might be a suitable method of testing protective strategies aimed at reducing the long-term adverse effects of TBI, such as novel helmet designs (Siegkas *et al.*, 2019). However, the model’s prediction needs to be validated against empirical data. This is difficult for human injuries because of the lack of precise biomechanical information of each injury. Experimental models of TBI give control of the impact biomechanics, especially when using electromagnetic impactors (Xiong *et al.*, 2013; Osier and Dixon, 2016*b*). This control provides the opportunity to compare the FE predictions with detailed neuroimaging and histopathological measures produced by the model.

Here, we developed a new high-fidelity FE model of the rat brain and simulated an injury mimicking the biomechanics of controlled cortical impact (CCI). FE modelling was used to predict strain and strain rate in grey and white matter following simulated mild and moderate impacts. Strain measures were then calculated in different brain regions, providing measures of deformation and its rate under impact loading. Both measures have previously been shown to predict grey and white matter injuries and blood–brain barrier damage after TBI (Shreiber *et al.*, 1999*a*, *b*; Bain and Meaney, 2000; Elkin and Morrison, 2007). Computational predictions were compared to high-field (9.4 T) MRI measures of traumatic axonal injury (TAI) and histopathology measures following CCI in rats.

MRI, specifically diffusion tensor imaging (DTI), is widely used to assess TAI in the subacute and chronic phase after head injury, as demonstrated by changes in different measures of diffusivity (Edlow *et al.*, 2016; Newcombe *et al.*, 2016). However, it is unclear whether these abnormalities are directly related to the immediate shear forces thought to initiate TAI. We investigated this by testing whether strain measures from our FE model predicted diffusion abnormalities in the white matter. The corpus callosum is the most commonly injured tract, as detected in human DTI studies and located beneath the impact site in our rat model. Hence, we focused analysis on this white matter structure.

Several diffusion MRI measures have been shown to relate to TAI in animal models of TBI (Mac Donald *et al.*, 2007*a*, *b*; Bennett *et al.*, 2012; van de Looij *et al.*, 2012). We focused on the most commonly used measures [fractional anisotropy (FA) and mean diffusivity (MD)], but also used multi-shell diffusion to perform neurite orientation dispersion and density imaging (NODDI), which is thought to provide information about cellular loss (neurite density) and axonal derangement (orientation dispersion) (Zhang *et al.*, 2012). This potentially provides a more detailed description of microstructural changes, which we tested by relating imaging changes to associated histopathology in the same location.

To validate the FE predictions and investigate the basis for neuroimaging abnormalities in the white matter, we performed immunostaining of microglial and astrocytic cells and neurofilaments along with histology of the corpus callosum integrity. Quantitative analysis was performed using a novel software-based image segmentation to identify individual cells and their morphological parameters in the corpus callosum. This has several advantages, including improved accuracy and lower bias, as compared to traditional thresholding or manual counting methods (Jaraj *et al.*, 2009; Johnson and Walker, 2015), allowing the relationship of strain and the post-injury pathology to be investigated with higher sensitivity. We specifically tested the hypotheses that mechanical strain immediately after impact would predict patterns of axonal injury seen in the corpus callosum, as measured by diffusion MRI, the number of glia cells and quantification of neurofilament staining within the same structure. This allowed us to test the relationship between predicted mechanical strains from a computational model and empirical measures of post-traumatic pathology.

## Material and methods

### Finite element modelling

**Figure 1:**
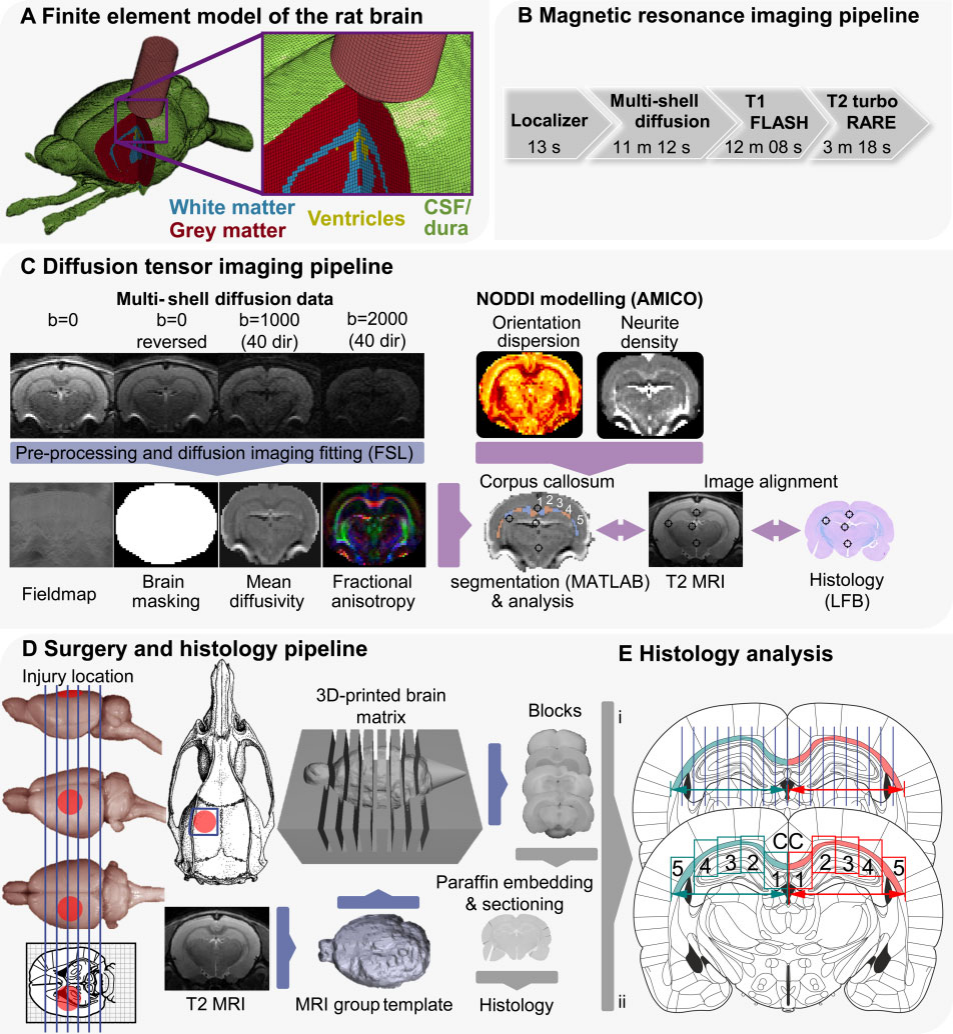
**Overview of methodology.** (**A**) The FE model of the rat CCI. The image shows CSF (green), grey matter (red), white matter (blue), ventricles (yellow) and impactor (pink). The skull and dura are not shown. (**B**) MRI pipeline: diagram showing the acquisition protocol. (**C**) DTI pipeline: flow chart of diffusion MRI image analysis. Following the acquisition of scans, all files were converted from Bruker format to NIfTI. Post-processing was performed with FSL tools topup, bet and eddy correct before independent simultaneous diffusion and neurite orientation dispersion and density imaging fitting (AMICO). The last stage involved image alignment with T_2_ MRI, histology and vice versa. This alignment was based on anatomical landmarks identified in the histology staining, MRI, the Paxinos and Watson, and Waxholm rat brain atlases. The corpus callosum was manually outlined and automatically segmented. A representation of the five segments obtained across the corpus callosum in each hemisphere is presented and comparable to **E**(**ii**). (**D**) Surgery and histology pipeline: approximate location of craniotomy and impact is shown on the rat skull and brain. Animals were subjected to either 1 (*n = *10) or 2 mm CCI (*n = *11.) From T_2_ images, a grouped 3D template was derived, which was 3D printed with 2 mm intervals. Blocks were cut from a selection of animals (1 and 2 mm CCI: *n = *6; naive/sham animals: *n = *7) using the matrix and one block (4, containing the core of the contusion) was selected for paraffin embedding. From paraffinized blocks, 7 µm sections were cut and every fifth section collected on slides (three per slide), therefore covering roughly 100 µm. (**E**) Sections were stained and analysed according to the described protocols and segments of the corpus callosum analysed using FIJI and HALO (analysis approach i: LFB; ii: microglia, astrocytes and neurofilaments). These sections were aligned with the MRI data, based on the procedures described in **C**. Rat brain, skull and atlas images from Paxinos and Watson (2007) and the University of Wisconsin-Madison Brain collection (http://neurosciencelibrary.org/Specimens/rodentia/labrat/index.html).

The high-fidelity FE model of CCI (Fig. 1A) was developed using the brain atlas of an adult male Sprague-Dawley rat (Papp *et al.*, 2014). The atlas was resampled using FMRIB to 160 µm voxel size and the FE mesh was developed with an in-house code, which uses the image-based meshing technique, thus allowing for the computational definition of the detailed anatomy of different tissues (Smith *et al.*, 2004; Ghajari *et al.*, 2017). Dura was defined with 20-µm thick shell elements and the skull was defined with rigid shell elements. A mesh smoothing filter was applied on the mesh at the surface of the model and the CSF/cortex interface. The final model consists of nearly 600 000 hexahedral elements and 100 000 shell elements, representing six different tissues, including grey matter, white matter, CSF, ventricles, dura and skull. We modelled the non-linear time-dependent mechanical behaviour of the brain with a nearly incompressible hyper-viscoelastic material model. Additional details are provided in the Supplementary material.

White matter mechanical properties were defined with an isotropic material model. There are conflicting reports regarding the anisotropy of the mechanical properties of the white matter. Some studies have reported mechanical anisotropy of porcine white matter under shear loading (Prange and Margulies, 2002; Ning *et al.*, 2006). However, recent work on human corpus callosum has not found mechanical anisotropy under shear, tension, compression and combined loading (Budday *et al.*, 2017), consistent with other experimental studies on porcine and bovine tissues (Nicolle *et al.*, 2004; Pervin and Chen, 2009). In addition, there is no previous work that has investigated mechanical anisotropy of the rat white matter. Hence, we modelled the rat white matter using an isotropic material model that is widely used for brain simulations.

Average displacement time history of the impactor was measured to be 3.5 m/s by high-speed videography, and this was used to define a constant velocity for the impactor across 1 mm and 2 mm indentations. The FE model was solved with a highly non-linear transient dynamic code, LS-DYNA (LS-DYNA Keyword User’s Manual, 2013).

### Animals and surgery

Experiments complied with a Home Office licence, the Animal (Scientific Procedures) Act 1986 and EU legislation. Male Sprague-Dawley rats (∼8–9 weeks, Charles River) were used, housed under standard conditions. All details on animal husbandry, randomization and blinding of investigators are described in the Supplementary material. After 1-week acclimatization, rats (*n = *18) were subjected to a baseline MRI scan.

General surgery and CCI procedure were carried out based on previous work (Donat *et al.*, 2007, 2016) with details and common data elements for CCI described in the Supplementary material (Smith *et al.*, 2015). Prior to surgery, animals were randomized into three groups: sham operation (*n = *3), mild/1 mm CCI (*n = *10) and moderate/2 mm CCI (*n = *11). Anaesthesia was induced with isoflurane and buprenorphine (subcutaneously) was used as perioperative analgesic. Body temperature was maintained at 37°C. A ∼6 mm unilateral rectangular craniotomy was performed, −0.5 mm to −6.5 mm posterior and +3.5 mm lateral to Bregma, (Fig. 1D), with the bone flap stored in sterile saline. Injury was induced with a flat steel impactor [5 mm diameter, ∼4 m/s, 1–2 mm depth, 100 ms contact time; Leica Impact One (Leica Microsystems)]. Based on previous classification and our MRI and histology data, injury would be classified as mild (1 mm) or moderate (2 mm) (Siebold *et al.*, 2018). Following impact, the dura was inspected for signs of rupture, which was found in one animal, where the craniotomy was only covered with absorbable gelatine sponges. On all other animals, the bone flap was reimplanted and sealed with a non-toxic light-curing resin (Technovit 2200; Kulzer). The incision was sutured, and the animals allowed to recover. Analgesia was given every 12 h (buprenorphine, per os) for at least 5 days. Sham-operated animals were subjected to all drugs and surgical procedures except craniotomy and impact. Additional naive animals (*n = *4) served as histology controls and were only subjected to a deep pentobarbital anaesthesia prior to transcardial perfusion. Fourteen days post impact, animals received a second MRI. One day later, rats were subjected to terminal anaesthesia, followed by transcardial perfusion and tissue harvest.

### Neuroimaging

MRI scanning was performed in a 9.4 T Bruker BioSpec scanner, equipped with a 4-channel phase array receiver coil and Paravision 6.1 software. MRI was acquired at two time points, pre and 2 weeks post surgery, using the pipeline shown in Fig. 1B.

Briefly, after localizer scans, high-resolution structural imaging was acquired with the following parameters: 3D T_1_ (echo time = 5 ms, repetition time = 60 ms, 0.2 × 0.2 × 0.2 mm^3^ resolution, 12-min acquisition) and 2D T_2_ (echo time = 33 ms, repetition time = 5.5 s, RARE factor = 8, 0.2 × 0.2 × 0.5 mm^3^ resolution, 20 slices, acquisition time ∼3 min). For diffusion, the multi-shell protocol included one shell with 40 gradient directions and b = 1000 s/mm^2^ and another with 40 directions and b = 2000 s/mm^2^. The protocol also contained four images without diffusion weighting (b = 0 s/mm^2^) and a single reversed phase encoding image without diffusion weighting. The EPI readout (echo time = 21 ms, repetition time = 4 s) had a resolution of 0.25 × 0.25 × 0.40 mm^3^. A total of 34 contiguous slices were acquired for whole brain coverage. The total scanning time for the multi-shell diffusion protocol was ∼11 min.

#### Structural MRI analysis

T_2_ and T_1_ images from the baseline time point were first combined to create a group template and the publicly available rat atlas (Waxholm Space Atlas) was then registered to the group template. FSL and ANTs were used for all affine and non-linear registration steps, respectively. Morphological distortions in the boundary of the cortex with the skull in the injured animals required masking the individual T_1_/T_2_ images, based on the previously created baseline group template. In addition, to remove the confounding effect of the hyper-intense lesions, semi-automatic segmentation using IMSEG v1.8 was conducted to delineate brain areas with focal lesions in the T_2_ images. The lesion masks were supplied as a weighting parameter to the final affine registration to group space. Additional information is provided in the Supplementary material.

#### Diffusion MRI analysis

The post-processing and analysis steps are shown in Fig. 1C. Correction of susceptibility induced distortions, eddy current distortions and rigid-body head motion was performed using FSL. Standard DTI metrics (FA, MD) were then extracted from the corrected multi-shell diffusion data using FSL dtifit. Neurite orientation dispersion and density imaging (NODDI) modelling was performed in parallel with the Accelerated Microstructure Imaging via Convex Optimization (AMICO) framework implemented in Python, which accelerates the fit up to four orders of magnitude by reformulating the model as a linear system, preserving accuracy and precision in the results. Metrics produced include neurite density and orientation dispersion.

The corpus callosum was chosen as a region of interest to assess the effects of CCI within the white matter. FSLeyes was used to manually draw binary masks of the corpus callosum in a single slice in the coronal view. Each mask was then automatically segmented into five equal sections using MATLAB (Fig. 1C). Segments closest to the midline were labelled as segment 1 and those furthest away were labelled as segment 5. Finally, masks were overlaid on DTI and NODDI images and average values within each specific segment were calculated.

### Histopathology and tissue staining

Brains were blocked, paraffin embedded, and serial coronal sections were cut from block 4 (Fig. 1D). Histopathology was performed in sham-operated/naive animals (*n = *7), mild CCI (*n = *6) and moderate CCI (*n = *6), with at least three sections per animal. To investigate general histopathological changes in the white matter and quantify the thickness of the corpus callosum, sections were stained with Luxol Fast Blue (LFB) and periodic acid-Schiff. Additional sections were immunofluorescently labelled for neurofilaments. Changes in distribution, number and morphology of glial cells were investigated by 3,3′-diaminobenzidine (DAB) immunostaining for microglia (IBA1) and astrocytes (GFAP) (details in the Supplementary material).

#### Image acquisition, histology quantification and MRI co-localization

Slides for light microscopy (LFB, IBA1 and GFAP) were imaged at ×20 with a slide scanner (Zeiss Axioscan Z1 with a Plan-Apochromat 20×/0.8 NA, Zeiss). Immunofluorescence was imaged at ×10 using a Zeiss Axio Observer Z1 (with a Fluar 10×/0.5 NA), with additional details described in the Supplementary material. On LFB stained slides, the corpus callosum was divided into five segments [Fig. 1E(i)] and thickness was measured every 500 µm. Two values were normalized to the corresponding contralateral segment of the same section and final values are expressed as per cent change of contralateral, with 100% being equal to the contralateral side. For neurofilament fluorescent staining, five equally-sized regions of interest (segments 1–5) were placed over the ipsilateral and the contralateral corpus callosum [Fig. 1E(ii); see Supplementary material for additional details]. Neurofilament fluorescence intensity was normalized to the corresponding contralateral segment of the same section and final values are expressed as per cent change of contralateral, with 100% being equal to the contralateral side.

Advanced quantitative analysis for IBA1+ and GFAP+ cells was performed using the modified HALO^®^ microglia module. Regions of interest were placed over the ipsilateral and the contralateral corpus callosum similar as for neurofilament fluorescent staining. Final values are IBA1/GFAP+ cells/mm^2^, with cells classified as ‘activated’ if their process thickness was over 2.7 µm. Process area and length are reported in micrometres (Supplementary material).

Co-localization of histological and DTI data was performed using a region of interest-based approach. Briefly, approximate coordinates from the Paxinos rat brain atlas were used to guide the selection of the T_2_ and corresponding DTI slices, with the latter being compared to the Waxholm Space atlas. Our histology blocks were cut using a 3D printed brain matrix (Fig. 1D). The matrix was based on averaged MRI data from the animals used, which allowed us to cut blocks with high replicability. LFB and haematoxylin stained sections from block 4 were used to identify white matter structures (corpus callosum, internal and external capsule) and general anatomical landmarks (lateral, third and dorsal third ventricle). As these structures are easily identifiable in T_2_ and DTI images, they were used to align both MRI and histology. Such a region of interest-based approach is often used in rodent models of TBI (Wang *et al.*, 2013*b*; Long *et al.*, 2015; San Martín Molina *et al.*, 2020).

### Statistical analyses

The effects of impacts on DTI measures of the individual corpus callosum segments were tested using a repeated two-way ANOVA, with segment and hemisphere as factors, followed by Sidak’s *post hoc* test. Histopathology data were analysed by two-way ANOVA with Tukey’s *post hoc* test. Factors were segment and impact. DTI and histopathology data are presented as the mean ± standard error of the mean (SEM). Predicted strain and strain rate data are presented as mean ± standard deviation (SD) in each segment.

We constructed linear mixed effects models to investigate the relationship between DTI (FA, MD, orientation dispersion and neurite density) and histopathological (thickness reduction, neurofilament fluorescence intensity, IBA+, ‘activated' IBA1+ and GFAP+ cells) measures of injury and FE predicted strains and strain rate in ipsilateral corpus callosum segments. The dependent variable was the change in injury measure in ipsilateral compared to the contralateral data. For the DTI measures, we used the contralateral side of the same animal, as it was acquired in the same scanning session. For the histopathological measures, we used the mean of the contralateral data across the naive/sham animals. Models were checked for normality, homoscedasticity and collinearity. Where these checks were not passed, logarithmic transformation was applied to the data to treat the model. Strain, strain rate, injury severity and their interaction were the fixed effects investigated and animals and segments were included as random effects. A backward step-wise approach was used to select the simplest model (Cheng *et al.*, 2010). The models that converged were compared and the best and simplest model was selected. The following model metrics were used to determine the best model. We used marginal R^2^, which describes the proportion of the variance explained by the fixed effects, to determine how well the model predicts the given output. We also calculated predictive R^2^, which explains how well the model predicts future data, and compared it with marginal R^2^ in order to indicate the risk of overfitting.

### Data availability

The data of this study are available from the corresponding author on reasonable request.

## Results

### Strain and strain rate predictions of the finite element model of controlled cortical impact

**Figure 2:**
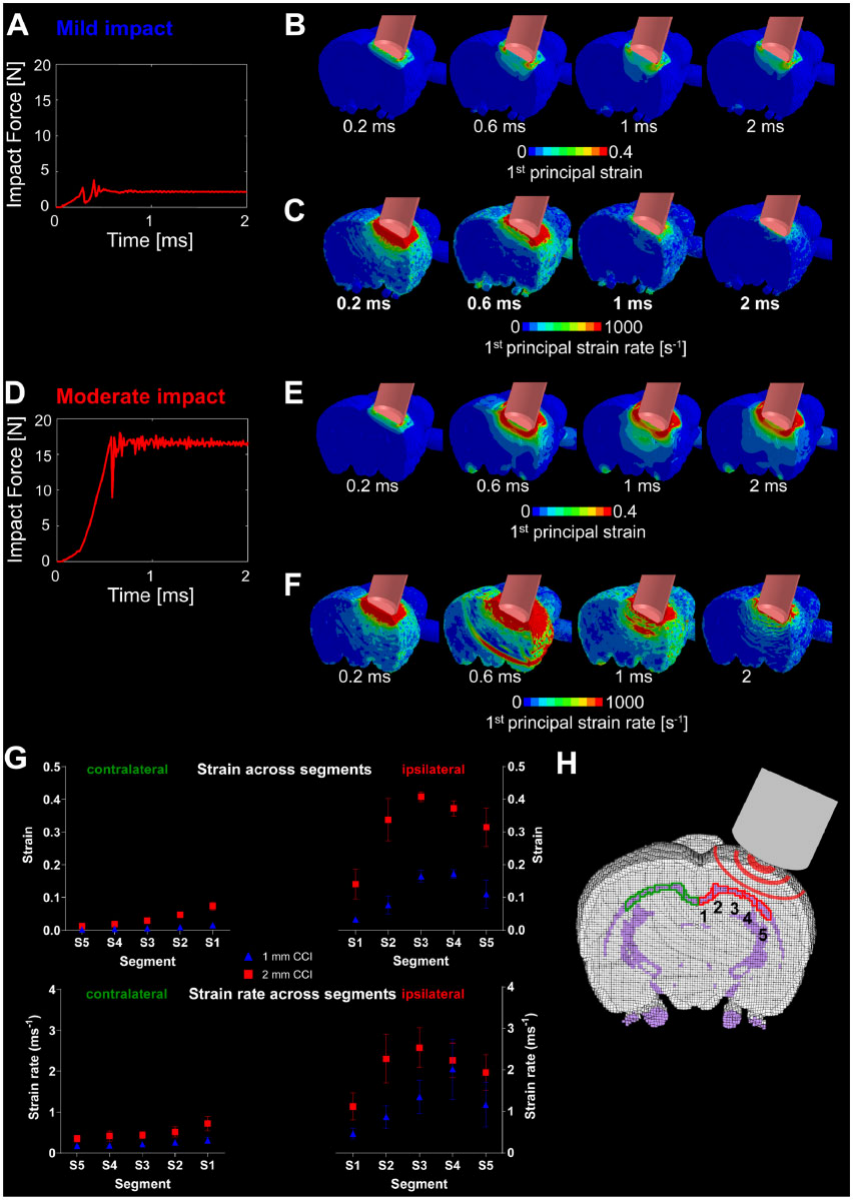
**Computational prediction of strain and strain rate following simulated impact.** (**A**) Impact force as shown over time for mild impact. (**B**) Time-variant first principal strain contour for mild impact. (**C**) Time-variant first principal strain rate contour for mild impact. (**D**) Impact force as shown over time for moderate impact. (**E**) Time-variant first principal strain contour for moderate impact. (**F**) Time-variant first principal strain rate contour for moderate impact. (**G**) Computational prediction of strain and strain rate in five segments of the corpus callosum at approximately −3.12 mm posterior to bregma. These correspond to the maximum value of strain and strain rate for each element throughout the simulation (see Fig. 3B, D and E). Data are presented as mean (± SD) strain/strain rate of the values in each segment. (**H**) A sketch of the five ipsi- and contralateral segments of the corpus callosum located at approximately −3.12 mm posterior to bregma.

The FE model predicted dynamic forces exerted on the dura and cortex (Fig. 2A and D), rapidly increasing during indentation. This was followed by oscillations due to the local motion of brain tissue before the force reached a constant value. The impact produced large strains and strain rates at the cortical impact site and deeper structures, including the corpus callosum and hippocampus (Fig. 2B, C, E and F). Increasing the indentation depth from 1 mm to 2 mm led to a 5-fold increase in the impact force (Fig. 2D), with large increases in strain and strain rates across a larger volume of the brain (Fig. 2E and F). Strain and strain rate were significantly larger in the ipsilateral segments of the corpus callosum (Fig. 2G). The highest values were predicted to occur in ipsilateral segments 3 and 4, located directly under the impactor. Significant strain and stain rates were also predicted in the contralateral corpus callosum, with the highest predicted for segment 1, the segment closest to the ipsilateral impact. Our model predicted an area of strain rate concentration far from the impactor (Fig. 2F at 0.6 ms). This is related to a wave of large particle velocity propagating through the brain tissue right after the impactor stops its motion (Supplementary Fig. 6). During the indentation, the impactor compresses the tissue underneath (blue area at 0.57 ms in Supplementary Fig. 6), leading to a similar vertical velocity of the tissue in its vicinity. When the impactor stops (time 0.58 ms), its velocity and the velocity of the tissue in its neighbourhood return quickly to zero. This sends a large wave of particle velocity back into the brain tissue, which shows highest concentration at the location where the ring is seen at 0.6 ms. This effect is not seen in the mild impact, which is likely to be because of the smaller indentation depth.

### Strain and strain rate predict the location of focal contusions

**Figure 3:**
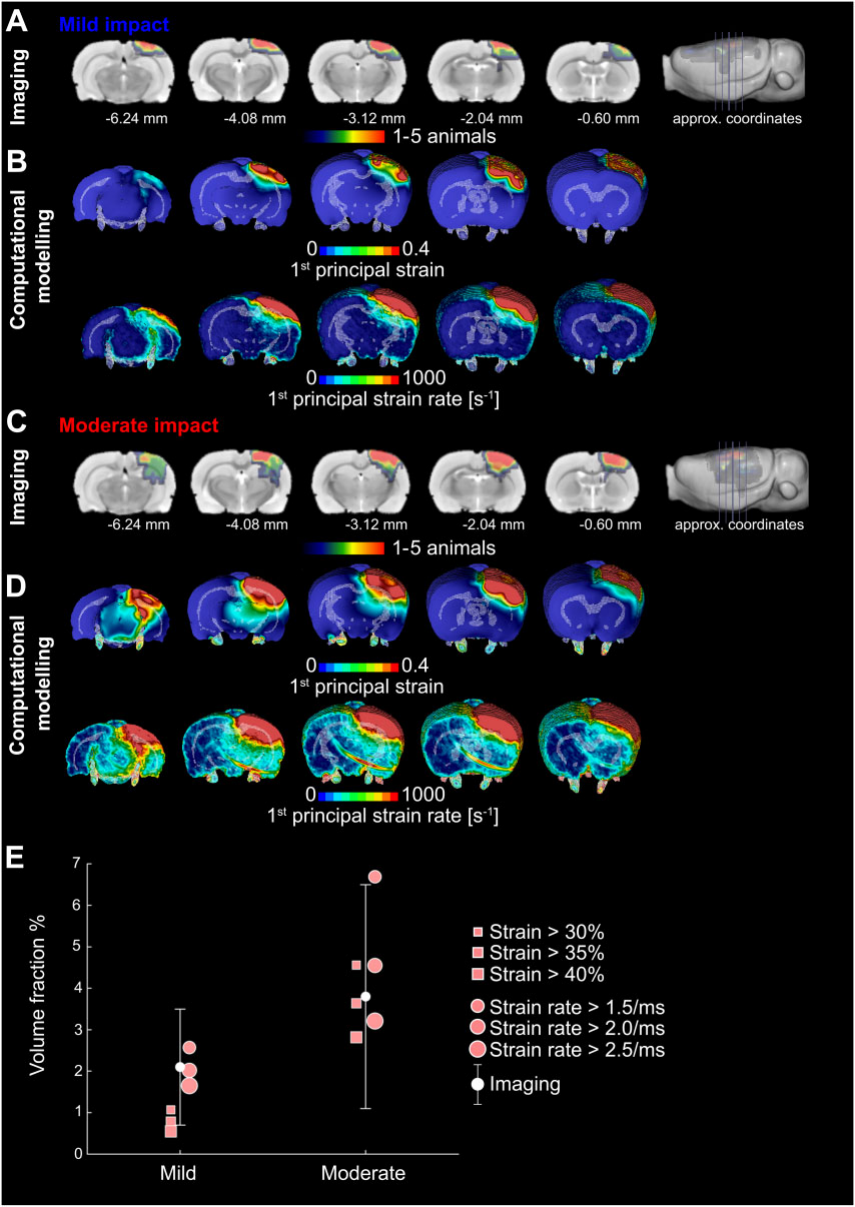
**FE modelling predicts contusion as measured by T_2_ MRI.** (**A**) Lesion probability maps after mild impact showing contusion/oedema with approximate coordinates from bregma. Colour scale indicates number of animals with visible lesions in T_2_-weighted images. Red–orange indicates regions where lesions were present in four or five (∼50%) of the CCI rats, green indicates regions where they were present in two or three (∼25%) and blue where a lesion was found in one post-surgery rat only; numbers indicate approximate coordinates from bregma. (**B**) First principal strain and strain rate predictions of the finite element model for mild injuries. These correspond to the maximum value of strain and strain rate for each element throughout the simulation. (**C**) Lesion probability maps after moderate injury showing contusion/oedema. (**D**) First principal strain and strain rate predictions of the finite element model for moderate injuries. These correspond to the maximum value of strain and strain rate for each element throughout the simulation. (**E**) Imaging: mean and standard deviation of the brain volume with contusion normalized by the total brain volume (volume fraction). These data are obtained from T_2_ lesion maps. The figure also shows the computational predictions of the volume of the brain that exceeds different values of strain and strain rate. The figure shows that the model prediction of the contusion volume is within one standard deviation of the empirical data.

Focal lesion maps derived from T_2_ neuroimaging were overlapped to produce a probability map for the location of lesions (Fig. 3A and C). These were then compared with computational predictions of strain and strain rates (Fig. 3B and D). As expected, focal damage was located in the region directly beneath the impact, extending deep into the cortical layers. High strains and strain rates were predicted in a similar location by the FE model. To make a quantitative comparison between MRI data and FE predictions, we calculated a contusion volume fraction by dividing the contusion volume by the brain volume. We also determined the volume fraction of brain exceeding strain values of 0.3, 0.35 and 0.4 and strain rate values of 1.5, 2.0 and 2.5/ms (Fig. 3E). Our results show that all but one strains, and all strain rates predict a lesion volume that falls within 1 SD of the mean value of the lesion volumes across all animals and both severities. This is in keeping with previous computational work (Mao *et al.*, 2006) and shows a reasonable prediction of lesion size from our computational model. The root mean square error of FE prediction of the contusion volume fraction versus mean value of experimental results was determined and provided evidence that a strain threshold of 0.3 and a strain rate threshold of 2.5/ms better predict the contusion volume.

### Diffusion tensor imaging provides evidence for white matter damage in the corpus callosum

**Figure 4:**
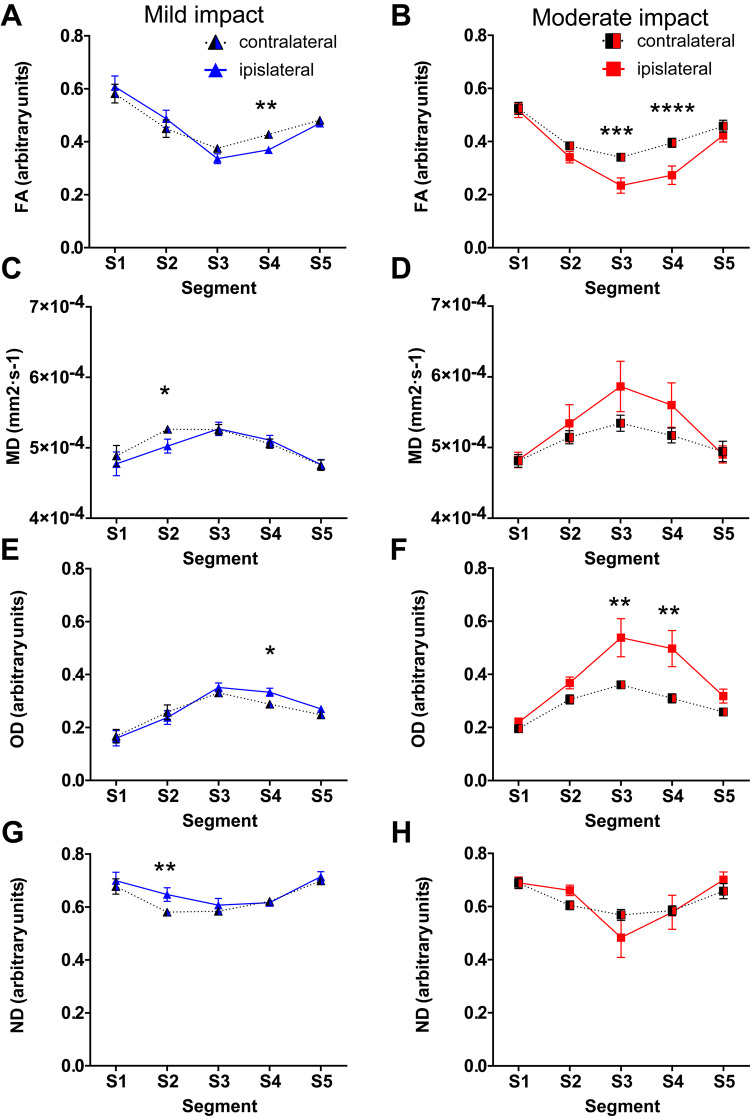
**Diffusion tensor imaging measures show white matter damage in corpus callosum segments subjected to highest strain.** Diffusion tensor imaging measures in segments of the corpus callosum across the ipsilateral and contralateral hemispheres. Fourteen days after mild/moderate impact, mean values of: (**A** and **B**) FA; (**C **and **D**) MD; (**E** and **F**) Orientation dispersion (OD); and (**G** and **H**) Neurite density (ND). All data are presented as mean ± SEM. Mild impact: *n = *10, moderate CCI: *n = *11. *P < 0.05, **P < 0.01, ***P < 0.001 and ****P < 0.0001 as compared to the contralateral side.

To quantify the white matter changes *in vivo*, diffusion measures were calculated from the corpus callosum segments (Fig. 4; entirety of the corpus callosum provided in Supplementary Fig. 2). Repeated measures two-way ANOVA was performed with segment and hemisphere as factors and *post hoc* tests comparing ipsilateral to contralateral side.

In the mild impact animals (Fig. 4A), ANOVA for FA showed a significant interaction between hemisphere and corpus callosum segment [*F*(4,45) = 7.960, *P < *0.0001]. *Post hoc* tests indicated a significant reduction in FA within segment 4 in injured animals [*t*(45.00) = 4.005, *P = *0.0011]. Reductions in FA within segments 2 and 3 were of borderline significance [*t*(45.00) = 2.584, *P = *0.0637 and *t*(45.00) = 2.680, *P = *0.0502, respectively]. Following moderate impact (Fig. 4B), ANOVA revealed a significant interaction between hemisphere and segment [*F*(4,50) = 3.786, *P = *0.0091]. *Post hoc* analysis revealed that reductions of FA in ipsilateral segments 3 and 4 were significantly different [*t*(50.00) = 4.215, *P = *0.0005 and *t*(50.00) = 4.825, *P < *0.0001] from corresponding contralateral segments.

For MD following mild impact (Fig. 4C), ANOVA showed a significant interaction between segment and hemisphere [*F*(4,45) = 2.620, *P = *0.0473]. *Post hoc* test revealed that MD values were significantly lower in ipsilateral segment 2 when compared to the contralateral side [*t*(45.00) = 3.259, *P = *0.0106]. In animals subjected to moderate impact (Fig. 4D), there was a significant main effect of hemisphere [*F*(1,50) = 4.829, *P = *0.0326] and segment [*F*(4,50) = 4.144, *P = *0.0056], but no interaction. *Post hoc* test showed no differences between ipsilateral and contralateral side.

For orientation dispersion following mild impact, there was a significant interaction of segment and hemisphere [*F*(4,45) = 3.558, *P = *0.0132] (Fig. 4E). This was due to significantly increased orientation dispersion only in segment 4 [*t*(45.00) = 3.278, *P = *0.0101] compared to the contralateral side. After moderate impact (Fig. 4F), there was a significant main effect of segment [*F*(4,50) = 16.15, *P < *0.0001] and hemisphere [*F*(1,50) = 20.06, *P < *0.0001], but no significant interaction. *Post hoc* test revealed that the changes in orientation dispersion were significantly different in segments 3 and 4 [*t*(50.00) = 3.479, *P = *0.0052 and *t*(50.00) = 3.676, *P = *0.0029] when compared to the contralateral side.

For neurite density after mild impact (Fig. 4G), there was a significant main effect of segment [*F*(4,45) = 6.901, *P = *0.0002] and hemisphere [*F*(1,45) = 10.07 *P = *0.0027], without an interaction. The increase in segment 2 was deemed significant in *post hoc* analysis [*t*(45.00) = 3.865, *P = *0.0018], when compared to the contralateral side. In animals subjected to a moderate impact (Fig. 4H), ANOVA showed only a significant main effect of segment [*F*(4,50) = 5.695 *P = *0.0007] without any significant changes in the ipsilateral segments when compared to the corresponding contralateral ones.

### The corpus callosum after controlled cortical impact

#### Moderate impact causes a marked thinning of the corpus callosum segments subjected to the highest strain

**Figure 5:**
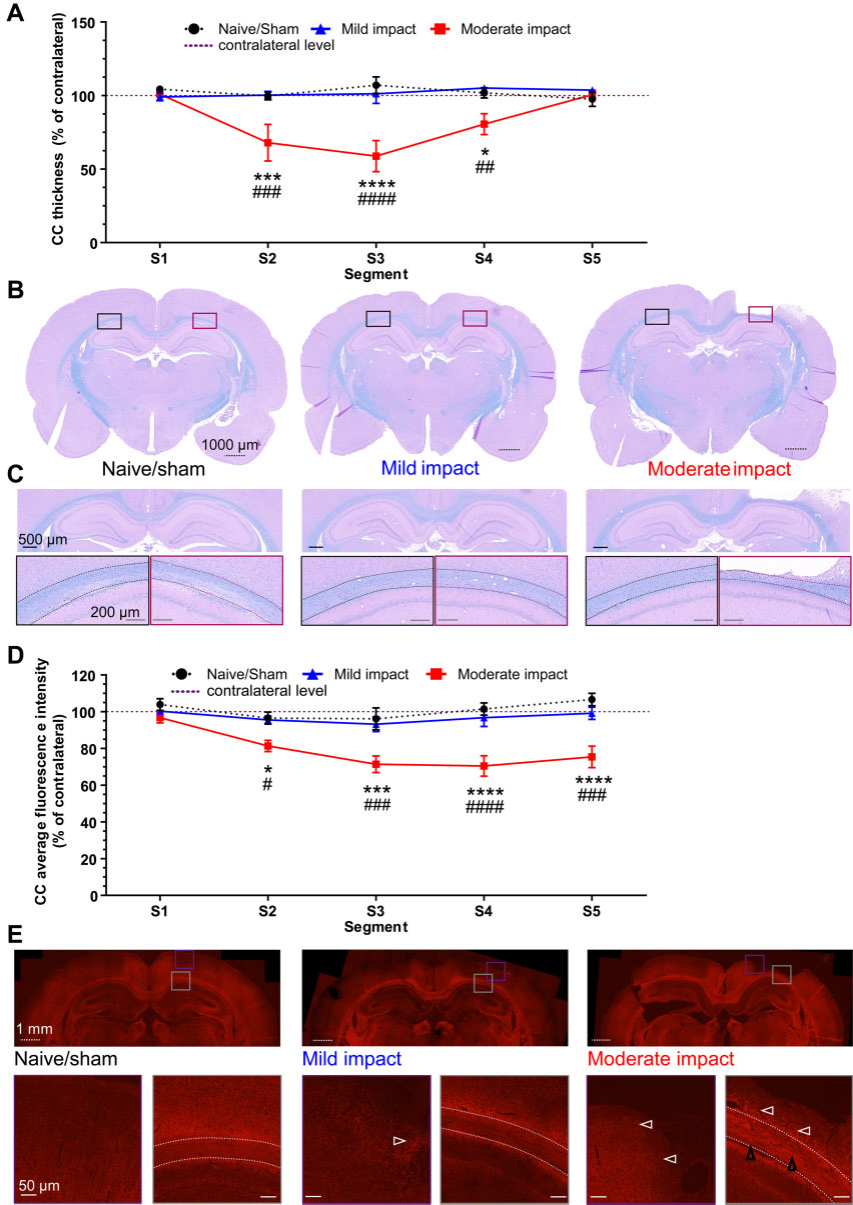
**Moderate impact causes thinning of corpus callosum in segments subjected to the high strain.** (**A**) Thickness quantification of individual segments of the ipsilateral corpus callosum (in % of the respective contralateral segment) as measured every 500 µm. (**B**) Representative whole brain photomicrographs of sections stained with LFB from naive/sham animals (*left*), mild impact (*middle*) and moderate impact. (**C**) The entirety of the analysed corpus callosum (dotted outline) of the respective groups, with coloured rectangles showing magnified views of the contralateral and ipsilateral corpus callosum. Black dotted scale bars = 1000 µm (whole brain sections); black solid lines = 500 µm (entire corpus callosum); grey solid lines = 200 µm (magnifications). All data are presented as mean ± SEM. Naive/sham: *n = *7; Mild impact: *n = *6, Moderate impact CCI: *n = *6. *P < 0.05, **P < 0.01, ***P < 0.001 and ****P < 0.0001 represent significant difference of moderate impact versus naive/sham animals. ^#^*P < *0.05, ^##^*P < *0.01, ^###^*P < *0.001 and ^####^*P < *0.0001) represent significant difference of moderate versus mild impact. (**D**) Quantification of Alexa 568 immunofluorescence intensity for neurofilament staining in five segments of the corpus callosum. (**E**) Neurofilament Alexa 568 immunofluorescence in naive/sham (*left* block), mildly injured (*middle* block) and moderately injured animals (*right* block). Coloured rectangles show magnified areas of the pericontusional cortex (purple) and CC segments (grey) below the impact, indicating axonal spheroid bulbs (white arrowhead), axonal swelling and disorganization (black arrowheads) prominently in moderately injured animals (*right* block). White dotted scale bars correspond to 1000 µm (half brain sections) and white solid lines to 50 µm (*insets*). White dashed lines indicate the outline of the corpus callosum. All data are presented as mean ± SEM. Naive/sham: *n = *5; mild impact: *n = *6, moderate impact: *n = *8. *P < 0.05, **P < 0.01, ***P < 0.001 and ****P < 0.0001 represent significant difference of moderate impact versus naive/sham animals. ^#^*P < *0.05, ^##^*P < *0.01, ^###^*P < *0.001 and ^####^*P < *0.0001 represent significant difference of moderate versus mild impact.

In naive/sham animals and those subjected to mild impact, LFB staining revealed no apparent differences between the hemispheres (Fig. 5B and C). In contrast, moderate impact resulted in a marked tissue loss and thinning of the corpus callosum (outlined area in Fig. 5C). A two-way ANOVA with segment and impact severity as factors, showed a significant interaction [*F*(8,80) = 4.506, *P = *0.0002]. *Post hoc* tests showed that corpus callosum thickness in the segments of the mild impact group was not significantly different to naive/sham animals. However, after moderate impact, the thickness across the three central segments (segments 2–4) was significantly reduced as compared to naïve/sham animals and mild impact animals (Fig. 5A).

Along with the general loss of corpus callosum structure in moderately injured animals, a reduction in fluorescence intensity of neurofilament staining also reflected the white matter damage. Following moderate impact, neurofilament fluorescence intensity in corpus callosum segments was found reduced upon visual inspection. Quantification of fluorescence intensity in the different segments of the corpus callosum supports this (Fig. 5D). Two-way ANOVA showed a significant main effect of impact [*F*(2,80) = 40.57, *P < *0.0001] and segment [*F*(4,80) = 4.204, *P = *0.0039], with an interaction not quite approaching significance [*F*(8,80) = 2.005, *P = *0.0562]. *Post hoc* analysis revealed that the normalized fluorescence intensity in segments 2–5 was significantly lower in animals subjected to moderate impact as compared to naive/sham and mildly injured animals. Qualitatively, axons and axonal bundles appeared disorganized and swollen (Fig. 5E, black arrowheads). Axonal spheroid bulbs (Fig. 5E, white arrowhead) were observed in the pericontusional cortex (Fig. 5E, insets) and prominently around the corpus callosum segments below the contusion (Fig. 5E, insets), indicating secondary axotomy or axonal loss. While some axonal spheroid bulbs were also found in the pericontusional cortex following mild impact (Fig. 5E, white arrowhead), the corpus callosum did not show the same changes as observed for moderately injured animals.

### The inflammatory response to injury in white matter is characterized by increased numbers of IBA1+ cells

**Figure 6:**
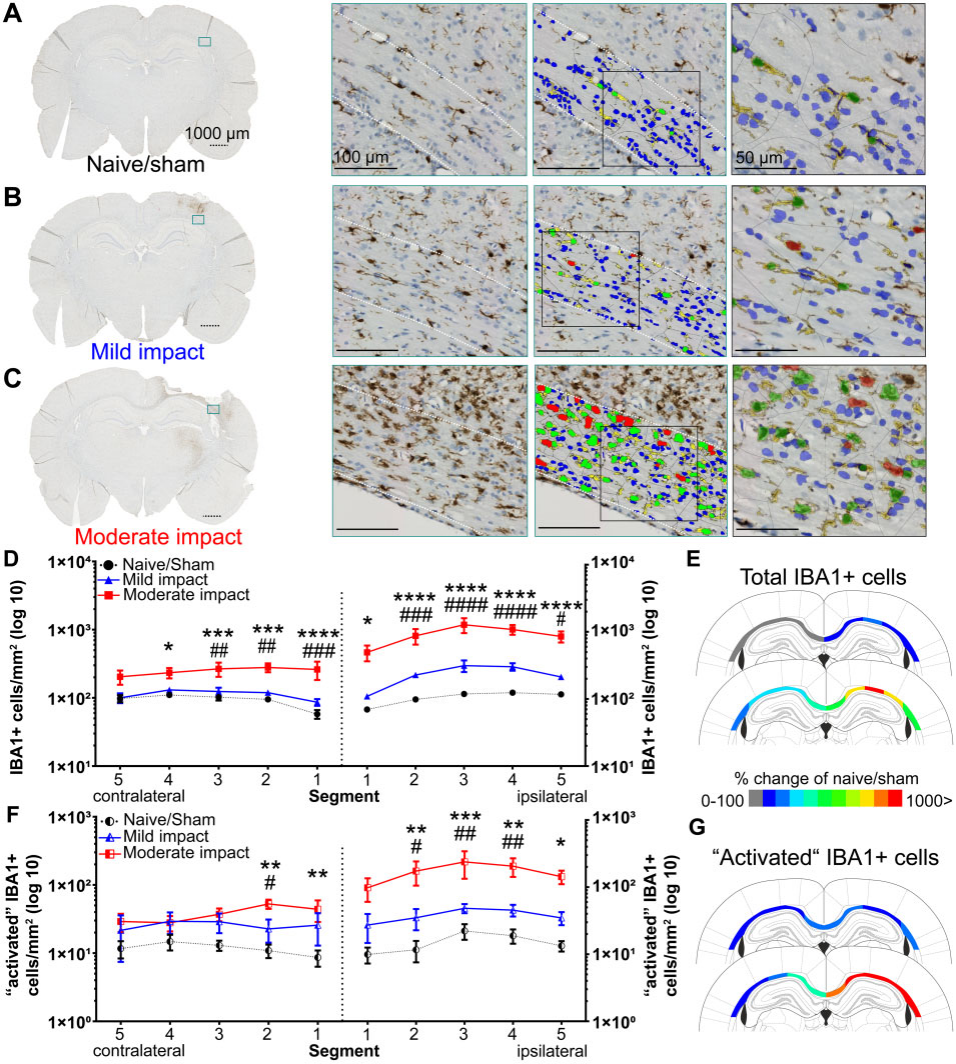
**Moderate impact causes a significant microglial response in the corpus callosum**. IBA1+ cells in the corpus callosum (dotted outline) of naive/sham animals (**A**) and following mild (**B**) and moderate (**C**) impact. Representative whole brain photomicrographs are shown in the *left* panel, with a coloured rectangle showing the magnified area (*middle left*). *Middle right*: The colour-coded overlay of detected IBA+ cells (green; red indicating ‘activated’ IBA+ cells) with processes (yellow) and haematoxylin+ cells not classified as microglia (blue). *Right*: A magnified view with transparent overlay. Dotted scale bars = 1000 µm, solid lines = 100/50 µm. (**D**) HALO quantification of total IBA1+ cells in five segments of the ipsilateral and contralateral corpus callosum. (**E**) Colour-coded heat map, showing per cent changes of total IBA1+ cells (rounded, compared to naive/sham animals) in the individual segments of the corpus callosum of animals subjected to mild (*top*) and moderate impact (*bottom*) (**F**) HALO quantification of IBA1+ cells classified as ‘activated' in five segments of the ipsilateral and contralateral corpus callosum. (**G**) Colour-coded heat map, showing per cent changes of IBA1+ ‘activated' cells (rounded, compared to naïve/sham animals) in the individual segments of the corpus callosum of animals subjected to mild (*top*) and moderate impact (*bottom*). All data are presented as mean ± SEM. Naive/sham: *n = *7; mild impact: *n = *6, moderate impact: *n = *6. *P < 0.05, **P < 0.01, ***P < 0.001 and ****P < 0.0001 represent significant difference of moderate impact versus naive/sham animals. ^#^*P < *0.05, ^##^*P < *0.01, ^###^*P < *0.001 and ^####^*P < *0.0001 represent significant difference of moderate versus mild impact.

Immunostaining of IBA1+ cells in the corpus callosum of naive/sham animals showed a morphology corresponding to a resting or low activity state, with small ellipsoid shaped cell bodies with fine processes, seemingly aligning with axonal tracts (Fig. 6A). Following impact, IBA1+ cells in the ipsilateral cortex and corpus callosum displayed enlarged somata, often with jellyfish or amoeboid morphology, including shorter and thicker or absent processes, indicative of a pro-inflammatory or activated phenotype (Fig. 6B, C, and Supplementary Fig. 3C). In addition, intermediate activation states were also observed, e.g. rod-like microglia (not shown).

Analysis of microglia density and distribution using HALO showed an increase in density of immunopositive cells in animals subjected to injury (Fig. 6D). ANOVA in the ipsilateral hemisphere, with impact and segment as factors showed a significant main effect of impact [*F*(2,80) = 67.33, *P *<* *0.0001] and segment [*F*(4,80) = 3.679, *P = *0.0084], but no interaction. Even though the number of IBA1+ cells were increased in each segment of the mild impact group, *post hoc* analysis indicated no statistical significance when compared with naive/sham animals. The main effects originate from the significant increase in density of IBA1+ cells in segments 1–5 of the moderate impact group, as indicated by *post hoc* analysis, when compared to both naïve/sham and mild impact animals.

Interestingly, the density of IBA1+ cells in the contralateral hemisphere was found to be increased in animals subjected to moderate impact when compared to the naive/sham and mild impact group (Fig. 6D). ANOVA showed a significant main effect of impact [*F*(2,80) = 34.65, *P < *0.0001]. *Post hoc* analysis revealed that the number of IBA1+ cells was higher following moderate impact as compared to naive/sham in segments 1–4 and mild impact in segments 1–3. No change in contralateral IBA1+ cell density was observed after mild impact, when compared to naive/sham animals.

When analysing the number of IBA1+ cells classified as ‘activated’ (Fig. 6F), ANOVA showed a significant main effect of impact [*F*(2,80) = 26.12, *P < *0.0001], but not of segment or an interaction. The effect of impact was primarily driven by an increase in density in segments 2–5 of animals subjected to moderate impact when compared to naive/sham animals, as shown by *post hoc* comparison. In the contralateral hemisphere, a significant main effect [*F*(2,80) = 12.84, *P < *0.0001] of impact, but not segment or interaction, on density of ‘activated’ IBA1+ cells was observed. This effect seemed to be confined to segments 1 and 2, as indicated by the *post hoc* comparison. The overall process morphology of IBA1+ cells in the corpus callosum consistently indicated an activated state, with shorter and thicker processes (Supplementary Fig. 3A–C), supported by the quantitative analysis. Detailed findings are described in the Supplementary material.

### Controlled cortical impact increases the number and morphology of reactive astrocytes in the corpus callosum

**Figure 7:**
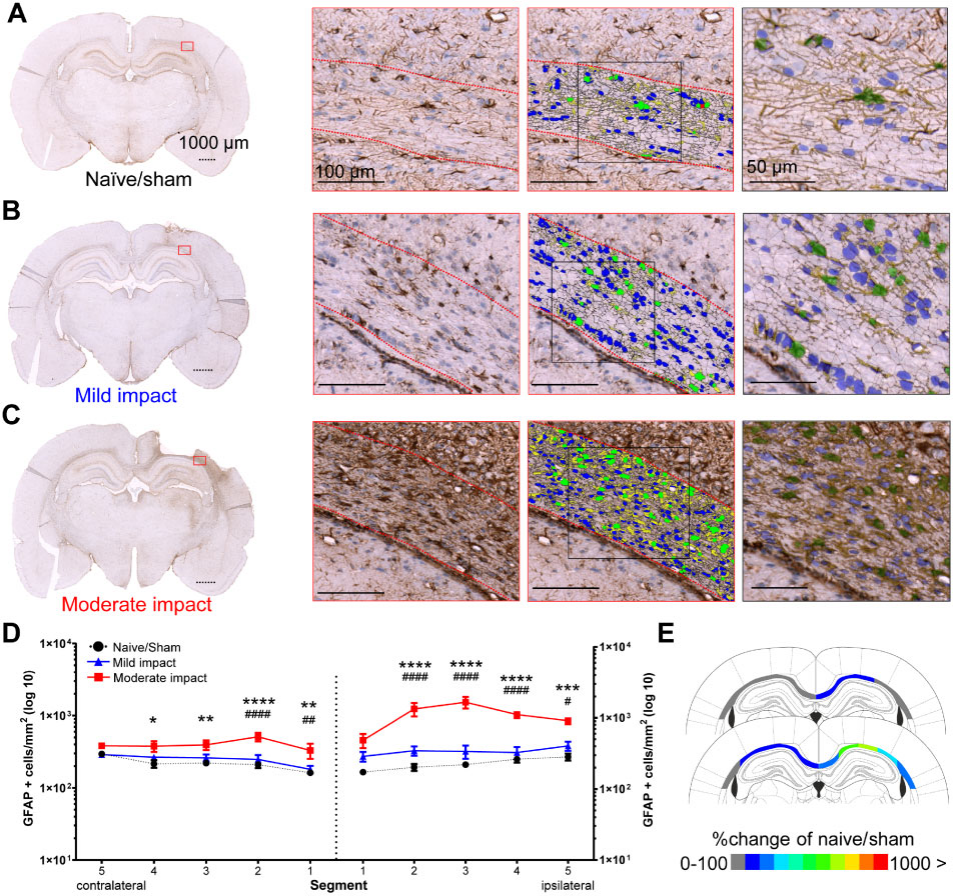
**Moderate impact causes a significant astrocytic response in the corpus callosum.** GFAP+ cells in the corpus callosum (dotted outline) of naive/sham animals (**A**) and following mild (**B**) and moderate (**C**) impact. Representative whole brain photomicrographs (*left*), with a coloured rectangle showing the magnified area (*middle left*). *Middle*: Colour-coded overlay of detected GFAP+ cells (green) with processes (yellow) and haematoxylin+ cells not classified as astrocytes (blue). *Right*: A magnified view with transparent overlay. Dotted scale bars = 1000 μm, solid lines = 100/50 μm. (**D**) HALO quantification of GFAP+ cells in five segments of the ipsilateral corpus callosum and one contralateral segment. (**E**) Colour-coded heat map, showing per cent changes of GFAP+ cells (rounded, compared to naïve/sham animals) in the individual segments of the corpus callosum after mild (*top*) and moderate impact (*bottom*). All data are presented mean ± SEM. Naive/sham: *n = *7; 1 mm impact: *n = *6, 2 mm impact: *n = *6. *P < 0.05, **P < 0.01, ***P < 0.001 and ****P < 0.0001 significance difference of moderate impact versus naive/sham animals. ^#^*P < *0.05, ^##^*P < *0.01, ^###^*P < *0.001 and ^####^*P < *0.0001) significant difference of moderate versus mild impact.

In naive/sham and mildly injured animals, GFAP+ astrocytes showed rounded to elongated cell bodies with thick processes, apparently aligned with axonal tracts in the corpus callosum (Fig. 7A and B). In contrast, astrocyte numbers were found to be increased in the ipsilateral corpus callosum of animals subjected to moderate impact. Particularly intense GFAP immunoreactivity was seen in segments 2–4, with immunopositive cells having larger somata (Fig. 7C) and extensive processes. Astrocytes showed highly ramified morphology with hypertrophic processes, especially in the vicinity of the contusion and areas of tissue loss of moderately injured animals, indicating the presence of the typical astrocytic scar.

Similar to the IBA1 analysis, the density of GFAP+ cells was quantified in the different corpus callosum segments using HALO (Fig. 7D). In the ipsilateral hemisphere, ANOVA indicated a significant main effect of impact [*F*(2,80 = 80.73, *P < *0.0001] and of segments [*F*(4,80) = 5.292, *P = *0.008], with a significant interaction [*F*(8,80) = 4.473, *P = *0.002]. The density of GFAP+ cells was significantly increased across segments 2–5 of animals subjected to moderate impact, compared to naive/sham animals and mild impact.

GFAP+ cell density was also increased in the contralateral hemisphere of animals subjected to moderate impact. ANOVA showed that the effects of impact [*F*(2,80) = 29.09, *P < *0.0001] and segments [*F*(4,80) = 3.064, *P < *0.0211] were significant, however, without interaction. *Post hoc* testing revealed that this was due to increased density in segments 1–4, as compared to naive/sham (segments 1–4) and mild (segments 1 and 2).

### Strain and strain rate are predictors of white matter imaging abnormalities and glia activation

We next investigated whether mechanical strain produced by the impacts predicts diffusion and histopathological abnormalities in the corpus callosum. Linear mixed effects models were used to investigate the relationship between multi-shell diffusion and quantitative histopathology measures and FE predicted strain and strain rate in ipsilateral corpus callosum segments.

#### Strain and strain rate decrease fractional anisotropy and increase orientation dispersion

**Figure 8:**
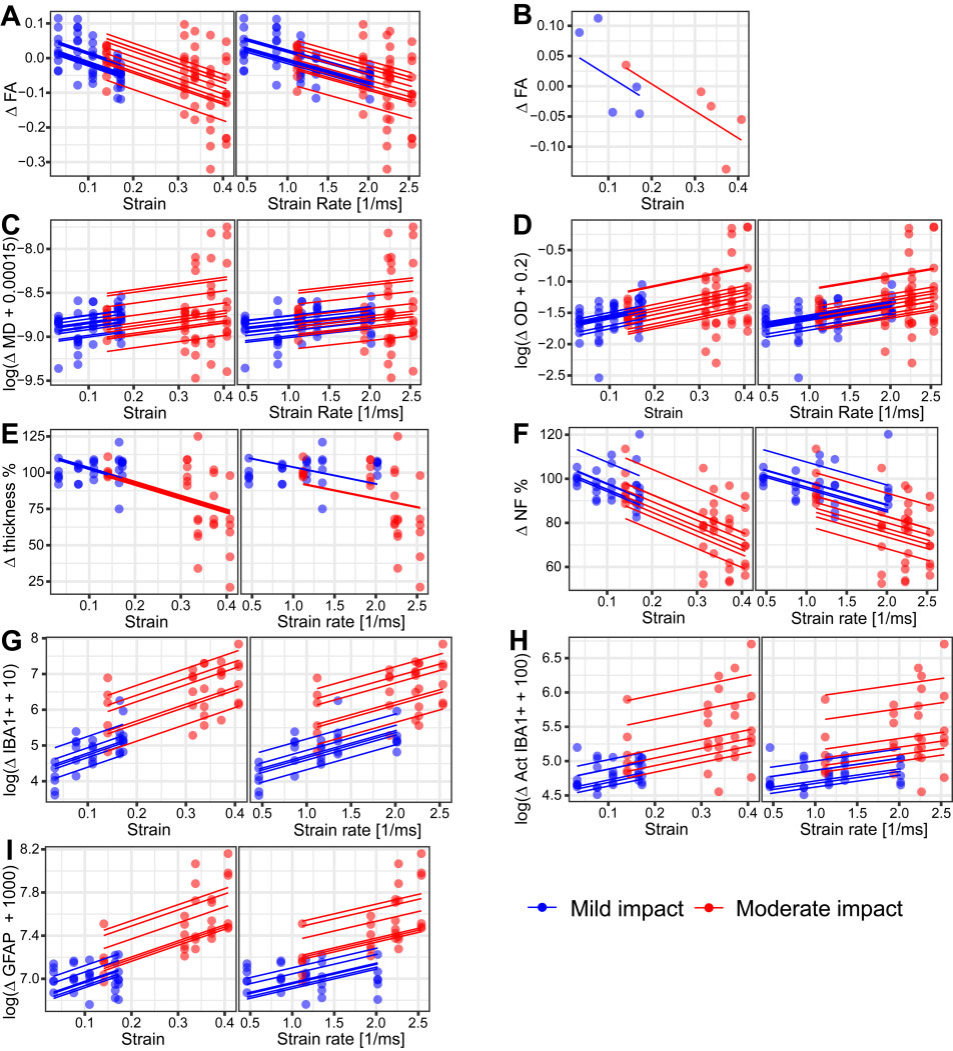
**Linear mixed effects model correlations of FE modelling predicted strain and strain rate with DTI and histopathology measures in the corpus callosum of animals subjected to impact.** Dots demonstrate experimental data (DTI and histopathology measures) in five segments of the corpus callosum. Solid lines exemplify the model predictions for individual subjects. Relationship of (**A**) FA and strain (*left*) and strain rate (*right*) for all animals. (**B**) An example of FA values in an animal subjected to mild and an animal subjected to moderate impact. Relationship of: (**C**) MD with strain (*left*) and strain rate (*right*); (**D**) orientation dispersion (OD) with strain (*left*) and strain rate (*right*); (**E**) corpus callosum thickness with strain (*left*) and strain rate (*right*); (**F**) Alexa 568 average immunofluorescence intensity for neurofilament staining with strain (*left*) and strain rate (*right*); (**G**) IBA1+ cells/mm^2^ with strain (*left*) and strain rate (*right*); (**H**) ‘activated’ IBA1+ cells/mm^2^ with strain (*left*) and strain rate (*right*); and (**I**) GFAP+ cells/mm^2^ with strain (*left*) and strain rate (*right*).

The pattern of strain within the corpus callosum predicted diffusion abnormalities seen at 14 days. Increasing strain was associated with reduced FA (Fig. 8A and B). A model including strain as the only fixed effect had a marginal R^2^ of 0.33 and a predictive R^2^ of 0.23, indicating that there was a risk of overfitting and that strain predicted 33% of the variation in FA. Adding severity as a second fixed effect reduced the marginal R^2^ to 0.29 but slightly increased the predictive R^2^ to 0.25, indicating a lower risk of overfitting. Increasing strain rate was also associated with reduced FA (Fig. 8A). A model with strain rate as the only fixed effect predicted 28% of the variation in FA, with a 0.28 marginal R^2^ and 0.25 predictive R^2^. Adding severity to this model as another fixed effect did not change its prediction. We also constructed a model with strain and strain rate as fixed effects. However, this model showed strong collinearity between predictors. Our further investigation showed a 0.96 Pearson’s correlation coefficient between strain and strain rate, which is expected based on the distribution of strain and strain rate across the segments of corpus callosum (Fig. 2G). Hence, we did not include both strain and strain rate as fixed effects in any mixed effects model.

Strain and strain rate predicted much smaller amounts of the variability in MD seen after CCI. A model with strain as a fixed effect had a marginal R^2^ of 0.08 and a much smaller predictive R^2^ of 0.03, indicating a risk of overfitting and that strain can only explain a very small portion of variance in MD in response to the injury (Fig. 8C). Adding severity to this model did not improve its predictions. Strain rate could also only explain a small portion of the variance (Fig. 8C). A model with strain rate as the only fixed effect had a marginal and predictive R^2^ of 0.06 and 0.03, respectively. Adding severity to this model did not improve its predictions. For neurite density, we could not find a transformation on the data that would lead to a model that passes the homoscedasticity and normality checks.

Increasing strain was associated with increases in orientation dispersion, calculated using NODDI (Fig. 8D). A model with strain as the only fixed effect had a marginal R^2^ of 0.22 and a predictive R^2^ of 0.17, indicating that 22% of the variation in orientation dispersion is explained by strain. Adding severity as another fixed effect did not improve the model prediction (0.20 marginal R^2^ and 0.16 predictive R^2^). Increasing strain rate was also associated with increases in orientation dispersion (Fig. 8D). A model with strain rate as the only fixed effect predicted 16% of the variation in orientation dispersion, with a 0.16 marginal R^2^ and 0.16 predictive R^2^. Adding severity to this model as another fixed effect increased the marginal R^2^ to 0.20 but the predictive R^2^ remained the same.

#### Strain, strain rate and impact severity decrease white matter integrity and increase the number of glia cells

Similar linear mixed effects modelling was used to explore the relationship between FE predicted strain and strain rate and quantitative measures of corpus callosum damage and associated glial activation. Increasing strain was associated with a decrease in corpus callosum thickness (Fig. 8E). A model including strain as the only fixed effect predicted 35% of the variation in the corpus callosum thickness due to the impact, with a marginal R^2^ of 0.35 and a predictive R^2^ of 0.29. Adding severity to this model as another fixed effect did not improve the prediction. A model with strain rate as the only fixed effect predicted 26% of the variation in the corpus callosum thickness, with a 0.26 marginal and 0.21 predictive R^2^ (Fig. 8E). Adding severity to this model as another fixed effect improved its prediction to 31%, with a marginal R^2^ of 0.31 and predictive R^2^ of 0.23.

Closely related to white matter structural changes, increased strain was also associated with a decrease in neurofilament fluorescence intensity (Fig. 8F). A model including strain as the only fixed effect predicted 51% of the variation in the neurofilament fluorescence intensity due to the impact, with a marginal R^2^ of 0.51 and a predictive R^2^ of 0.47. Adding severity to this model as another fixed effect did not improve the prediction. A model with strain rate as the only fixed effect predicted 29% of the variation in the neurofilament fluorescence intensity, with a 0.29 marginal and 0.36 predictive R^2^ (Fig. 8F). Adding severity to this model as another fixed effect improved its prediction to 44%, with a marginal R^2^ of 0.44 and predictive R^2^ of 0.39.

Increasing strain was also associated with an increase in the number of IBA1+ cells (Fig. 8G). The model including strain as the only fixed effect was able to predict 51% of the variance and it had a very low risk of overfitting with a 0.67 predictive R^2^ versus 0.51 marginal R^2^. Adding severity as another fixed effect increased marginal R^2^ to 0.73, but it did not improve the predictive R^2^. The model with strain rate and severity as fixed effects was able to predict 73% of the variance, with a 0.73 marginal R^2^ and 0.69 predictive R^2^ (Fig. 8G). Removing severity from this model reduced the marginal R^2^ to 0.30 (0.55 predictive R^2^).

Increasing strain also increased the number of IBA1+ cells classified as ‘activated’ (Fig. 8H). The model including strain as the only fixed effect had a 0.21 marginal R^2^ (0.35 predictive R^2^), which means strain can explain 21% of the observed microglial activation. Adding severity as another fixed effect increased marginal R^2^ to 0.44 and predictive R^2^ to 0.37, indicating a slight risk of overfitting. We made a different observation for strain rate (Fig. 8H). The model with strain rate as the only fixed effect had a marginal R^2^ of 0.08 (0.24 predictive R^2^) and adding severity to this model significantly improved the marginal R^2^ to 0.42, with a predictive R^2^ of 0.36.

Increasing strain resulted in larger numbers of astrocytes in corpus callosum (Fig. 8I). The model including both strain and severity as fixed effects predicted 65% of the variation in the number of astrocytes, with 0.65 marginal R^2^ and 0.61 predictive R^2^. Removing severity from this model lowered the marginal R^2^ to 0.52 (0.59 predictive R^2^) and removing strain from the model reduced the marginal R^2^ to 0.54 (0.50 predictive R^2^). Increasing strain rate also increased astrocytes in corpus callosum (Fig. 8I). The model including both strain rate and severity as fixed effects predicted 61% of the variation (0.61 marginal and 0.56 predictive R^2^). Removing severity from this model lowered the marginal R^2^ to 0.23 (0.43 predictive R^2^).

## Discussion

This study shows that the predictions of our computational model of injury biomechanics correlate with *in vivo* MRI measures of axonal injury, quantification of neurofilament fluorescence intensity and the glial response, including morphology, as produced by a rat CCI model. The CCI model was chosen as all biomechanical parameters can be quantitatively defined with a high level of reproducibility (Osier *et al.*, 2015; Osier and Dixon, 2016*a*, *b*). This control allows for a more precise definition of the biomechanical parameters in the FE model, as compared to other animal models of TBI, therefore facilitating a better understanding of the relationship between biomechanical parameters, predicted strain and *in vivo*/post-mortem end points. Our approach made it possible to link the immediate biomechanical effects to MRI measures of axonal injury, supported by quantitative post-mortem measurements of glia activation in the subacute period of TBI. We found a clear relationship between the immediate mechanical strain from impact and post-traumatic brain pathology at 2 weeks after impact, including corpus callosum MRI abnormalities, neurofilament fluorescence intensity and neuroinflammation. These pathologies are key features observed in human patients and regarded as important biomarkers, specifically blood levels of neurofilaments (Kinnunen *et al.*, 2011; Ramlackhansingh *et al.*, 2011; Hernandez-Ontiveros *et al.*, 2013; Johnson *et al.*, 2013*a*; Zetterberg *et al.*, 2013; Svingos *et al.*, 2019), thus making our FE model a novel tool to predict the likelihood of neuropathology being produced by TBI.

Our high-fidelity biomechanics model of the rat brain allows a detailed prediction of forces in the whole brain. One novelty of our approach is that the finite element meshes representing the brain tissues were generated from a high-resolution atlas using an image-based meshing technique (Ghajari *et al.*, 2017). This allowed us to incorporate the detailed anatomy of different brain regions into the model. In addition, the mechanical response of the brain tissue was defined with a material model and properties that were able to predict shear stiffening of the brain tissue at high strain rates, as expected in the CCI experiments. The shear response of the brain tissue is highly dependent on the rate of deformation in a way that the stiffness of the tissue increases substantially when the rate of deformation is increased and accurate modelling of this effect is key to the prediction of strains (Nicolle *et al.*, 2004). Incorporating high rate mechanical properties and detailed anatomy of the brain into the model allowed us to accurately predict strain and strain rate distributions in key regions, particularly in the corpus callosum, where progressive axonal injury and neuroinflammation are seen after TBI (Smith *et al.*, 2003).

The brain tissue undergoes large strain and strain rate in the CCI experiments, which requires implementation of appropriate material behaviour in the computational model. Currently, there are no mechanical properties available for the rat brain that are suitable for the strains and strain rates seen in the CCI experiments. Hence, to model the rate sensitive response of the brain tissue at the very high rates seen in CCI, we adopted the shear relaxation modulus from the only experimental study that has extended the characterization of the human brain tissue to very high frequencies relevant to the CCI (Finan *et al.*, 2012). To account for the difference between human and rat brain, we scaled the relaxation modulus by using the ratio between the long-term shear modulus of the rodent cortex to that of the human. We used the same properties for the grey and white matter, because previous work has shown that the shear relaxation modulus early after indentation is nearly the same for these tissues in rat and in human (Nicolle *et al.*, 2004; Finan *et al.*, 2012). Future work, particularly *in vivo* techniques such as magnetic resonance elastography (Bayly *et al.*, 2012), may help to determine more accurate properties for different tissues in human and rodents.

The distribution of corpus callosum abnormalities correlates well with the strain and strain rate predictions, with white matter segments undergoing larger strains showing more pronounced abnormalities in several outcome measures. This is in line with previous work, showing that mechanical strain is a key initial factor in determining pathology after brain injury. For instance, dynamic stretching of the optic nerve of guinea pigs revealed a relationship between axonal damage and mechanical strain, with larger strains more likely to cause axonal swelling or retraction bulbs along the axons (Bain and Meaney, 2000). For the first time, we demonstrate this relationship in the corpus callosum, a major white matter tract, and determine a correlation between the spectrum of white matter damage, quantified by high-field diffusion MRI and immunostaining, and mechanical strain distribution. Diverse white matter damage and degeneration are commonly seen after TBI and in long-term survivors of TBI (Johnson *et al.*, 2013*a*; Sussman *et al.*, 2017). Our results indicate that strain distribution is a major factor in predicting the patterns of white matter injury, as shown by the quantitative loss of LFB-positive white matter structure and neurofilament fluorescence intensity. Furthermore, neurofilament staining indicated axonal swelling and disorganization along with axonal spheroid bulbs in moderately injured animals, along with a significant reduction in staining intensity. A strong linear relationship was found between strain and microglial activation, as measured by quantitative IBA1 staining in the corpus callosum. Maximum strain occurred within the first few milliseconds of the impact loading, but a large proportion of the variance in total microglia at 2 weeks post injury was explained by strain alone. This strong relationship is striking, considering the high complexity of the inflammatory response after TBI (Wofford *et al.*, 2019). Strain likely causes axonal membrane disruption, as demonstrated after closed-head injury, where only neurons showed uptake of a parenchymal dye (Wofford *et al.*, 2017). Together with DAMPs and pro-inflammatory cytokines, this then acts as driver of the glia response (Braun *et al.*, 2017).

Our findings that strain is a predictor of the glia response provides strong evidence for the validity of our FE model, as microglial activation is a major aspect of the neuroinflammatory response after TBI in humans and laboratory animals (Ramlackhansingh *et al.*, 2011; Loane *et al.*, 2014; Simon *et al.*, 2017). Microglia are furthermore implicated in secondary axonal injuries, either by specifically targeting injured axons following TBI and other lesions or responding with activation to the initial axonal damage (Bechmann and Nitsch, 1997; Wang *et al.*, 2013*a*; Lafrenaye *et al.*, 2015). The number of microglia in segments of the contralateral corpus callosum was increased in moderately injured animals, even though our model predicted only small strains. While at first seemingly contradicting, this seems to be a frequently observed secondary effect, corresponding to glia activation in more remote brain regions, e.g. the thalamus, not immediately after impact (3–7 days) but thereafter (Raghavendra Rao *et al.*, 2000; Donat *et al.*, 2016).

We observed a remarkable effect of injury severity on astrocytes, showing minimal changes in the corpus callosum after mild injury, but a significant increase after moderate injury. Strain acting directly on astrocytes might explain this, as there is *in vitro* evidence that mechanical forces can affect astrocytes directly, as indicated by release of DAMPs and other proteomic signatures (Levine *et al.*, 2016; Xiong *et al.*, 2018). However, no astrocytic membrane disruption was observed in the porcine closed-head model that showed membrane disruption in neurons (Wofford *et al.*, 2017). Another explanation might be that microglia activation after moderate injury, primarily explained by strain, also directly affects astrocytic activation. Recent data suggests crosstalk of astrocytes and microglia, which could potentially result in a specific neurotoxic phenotype of astrocytes (Villacampa *et al.*, 2015; Liddelow *et al.*, 2017). This is supported by a reported correlation of rod-like microglia and GFAP+ cells following fluid-percussion injury and microglial elimination attenuating astrogliosis, but not axonal injury (Witcher *et al.*, 2018).

Our *in vivo* MRI data showed that FA and orientation dispersion are the most sensitive measures to detect white matter changes after TBI, as exemplified by their effect sizes in the central segments. MD and neurite density only showed low effect sizes. Demyelination and/or axonal degeneration are generally attributed to MD (Johnson *et al.*, 2013*b*). Mac Donald *et al.* subjected mice to CCI of a similar severity to our moderate impact and reported a strong decrease in relative anisotropy around 40% of the pericontusional white matter, beginning at 4–6 h and lasting up to 1 month after injury, similar to our findings (Mac Donald *et al.*, 2007*a*). In contrast, when a milder injury is induced, only transient increases in FA are found at 7 days post injury, without any histopathological changes, and returning to sham levels at 14 days, more similar to our mild impact (Hoogenboom *et al.*, 2019).

While FA is sensitive to general white matter abnormalities, it is lacking specificity, mainly reflecting a combination of axon density, axon distribution, gliosis, oedema and degree of myelination. Other measures might be more specific indicators. NODDI potentially provides measures of higher biological specificity, with less bias from crossing fibres and excellent agreement with electron microscopy measures of fibre density (Sepehrband *et al.*, 2015; Kodiweera *et al.*, 2016). Orientation dispersion has been proposed as a more specific measure of microstructure with higher values in areas of crossing fibres compared to parallel fibres in different areas of the mouse brain (Sato *et al.*, 2017). In our study, increased orientation dispersion in the corpus callosum is associated with the high strains in the area. Both FA and orientation dispersion are assumed to reflect the actual structure of the white matter, with orientation dispersion offering the advantage of modelling axons and being less susceptible to partial volume effects from CSF and oedematous lesions.

Several limitations apply to our employed methodology. Computational predictions are only aligned with MRI and quantitative histology in a single block. However, as this block contains the contusion core, it can be hypothesized that our FE predictions of strain and strain rate are also applicable to the contusion and contusion borders in adjacent blocks. We have opted for a relatively thicker MRI slice compared to the in-plane resolution (0.25 × 0.25 × 0.40 mm^3^) to allow for a stronger diffusion signal collected in a shorter scan time. The use of isotropic voxels is recommended to ensure that the FA values measured in regions containing crossing fibres (as present in the cingulum in our slices) are not prone to more noise caused by the use of non-isotropic DTI (Oouchi *et al.*, 2007). Another limitation of this work is that only one time point was investigated. This complicates the direct connection of biomechanical tissue strain during the impact and markers of injury several days post impact. Future studies will therefore need to focus on investigating the temporal relationship of strain and white matter abnormalities by understanding how quickly mechanical strain elicits relevant *in vivo* and post-mortem changes in the white matter. While we focused our outcome measures on major histopathological changes, such as neurofilament levels, glia activation and translatable MRI, probing the direct relationship of strain and tissue damage immediately after injury would further help validating the FE model. While our optical imaging approach provides robust quantification of cell density, the quantification of glia process metrics is affected by some methodological restrictions. Using thin paraffin sections and lower-power magnification is likely not fully representative of the totality of microglia processes, e.g. very thin processes <0.5 µm. Tissue clearing and high-power confocal imaging in 3D would be more suitable to capture the entirety of the microglial arborization.

While the CCI model provides excellent biomechanical control and reproducibility, it usually causes less primary axonal injury compared to other animal models. Our staining, however, indicates a substantial loss of neurofilaments and axonal damage at ∼14 days post-injury in animals subjected to moderate impact, which is in line with previous studies using the CCI model (Dixon *et al.*, 1991; Smith *et al.*, 1995). Other animal models [e.g. fluid-percussion or rotational acceleration models such as CHIMERA (closed-head impact model of engineered rotational acceleration)] might be more suitable to investigate axonal injuries (Cheng *et al.*, 2019; Desai *et al.*, 2020). These models would allow a direct quantitative analysis of primary axonal damage in the first hours after injury and in turn a better comparison to the strain predictions, as both seem tightly connected directly after injury. Quantification of other early markers of cellular injury, e.g. DAMPs, such as HMGB1 or extracellular adenosine/ATP, that are released within minutes after injury, could also provide a higher temporal resolution to image cellular damage patterns in response to strain (Wofford *et al.*, 2019).

One potential limitation of our work lies in the differences in brain structure between rodents and humans. The lissencephalic structure of the rodent brain could limit the applicability of our findings to effects that depend on the presence of sulci, which are absent in the rat. This is most likely to be problematic for the study of chronic traumatic encephalopathy pathology, which accumulates at the depths of the sulci. This is not the focus of our research and does not directly impact on the observations we have made about the relationships between biomechanical forces and glial response and axonal injury. However, further work with gyrencephalic animals such as ferrets or pigs would allow the impact of sulcal anatomy on the relationship between biomechanical forces and brain injury to be studied directly (Schwerin *et al.*, 2017, 2018; Hutchinson *et al.*, 2018).

Using the rat model along with the computational prediction of the distribution of mechanical forces allowed us to determine the relationship between strain and strain rate and pathology. Although the direct translation of the reported correlations and thresholds from rodents to humans is limited due to the differences in brain morphology, this study validates the use of strain and strain rate in computational models of human TBI. This validation provides measures of the mechanical forces that should be reduced by protection strategies, in order to mitigate the acute and long-term effects of TBI. Determining correlations between force, pathology and injury thresholds in the human brain remains a key challenge for future work, not least because the initial loading often remains unknown. Accurate measurements of the head motion by using video analysis or head-mounted sensors can yield the loading conditions required to inform computational models of TBI, which in turn allows to predict the distribution of mechanical forces in the brain. Correlating force distribution with patterns of pathology, mapped from clinical or post-mortem assessments, can be then used to determine thresholds for mechanical forces that produce acute and long-term damage in the human brain.

A validated high-fidelity finite element model of TBI is a unique tool that predicts pathology in different tissues and anatomical regions by using a mechanical description of the injury, such as the head motion in the few milliseconds of the injury. Key applications of this tool will be in predicting the pathological sequelae of head injuries due to different injury patterns and how this could drive neurodegenerative processes. Furthermore, it can be used to evaluate the protection effects of TBI prevention technologies, such as helmets and airbags. Current predictive measures of TBI, such as linear acceleration of the head or g force, disregard the complex anatomy of the brain and its interaction with mechanical forces in producing different pathologies with distinct outcomes. The strong evidence that connects the predictions of our finite element model of TBI to the *in vivo* and post-mortem outcome measures allows us to predict patterns of brain tissue damage, particularly in key regions such as sulci and white matter tracts. This approach has the potential to improve injury assessment methods and protective equipment designs in order to effectively predict and prevent TBI and its associated progressive pathologies.

## Supplementary Material

awaa336_Supplementary_Data

## Abbreviations

CCI =: controlled cortical impact
DTI =: diffusion tensor imaging
FA =: fractional anisotropy
FE =: finite element
MD =: mean diffusivity
NODDI =: neurite orientation dispersion and density imaging
TBI =: traumatic brain injury
