# Supplementary material for: From biomechanics to pathology: predicting axonal injury from patterns of strain after traumatic brain injury
Source: Brain. 2021 Jan 17;144(1):70–91. doi: 10.1093/brain/awaa336 (PMC7990483; doi:10.1093/brain/awaa336)
Supplement: awaa336_Supplementary_Data [file awaa336_supplementary_data.zip › brain-2020-00063-File010.pdf]

Supplementary material

1 Finite element modelling

The volume preserving response of the brain tissue was modelled with a hyper-viscoelastic material model that combines the Ogden hyperelastic model with the linear viscoelastic model:

$$\mathbf{S}(t) = \mathbf{S}^{\infty} + \int_0^t G(t-T)\frac{\partial \mathbf{E}(T)}{\partial T}dT\,. \qquad (2)$$

Here $\mathbf{S}(t)$ is the time-dependent second Pialo-Kirchhoff stress tensor, $\mathbf{E}$ is the Green-Lagrange strain tensor and $\mathbf{S}^{\infty}$ is the equilibrium state second Pialo-Kirchhoff stress tensor, obtained from the Ogden hyperelastic model with a stain energy function given by:

$$\Psi^{\infty} = \sum_{p=1}^{n} \frac{\mu_p}{\alpha_p}\left(\lambda_1^{\alpha_p} + \lambda_2^{\alpha_p} + \lambda_3^{\alpha_p} - 3\right). \qquad (1)$$

λ_1, λ_2 and λ_3 are principal stretches of the brain tissue, and μ_p and α_p are material constants. $G(t)$ is the relaxation function represented with a Prony series of the form:

$$G(t) = \sum_{i=1}^{n} G_i e^{\frac{-t}{\tau_i}}\,, \qquad (3)$$

where τ_i and G_i are material constants.

The incompressible volumetric response of the tissue was modelled with a large bulk modulus. The volume preserving (shearing) response of the brain tissue was modelled with a hyper-viscoelastic material model. The craniotomy was modelled by removing the skull elements and the impactor was modelled with rigid shell elements.

There are limited experimental data on mechanical properties of rat brain at high loading rates, in contract to the human brain. Previous work performed indentation tests on adult rat brain (Finan *et al.*, 2012). However, the loading time was 70ms, which is significantly longer than the impact time in the CCI model, and the tissue was subjected to small strains while in CCI the tissue undergoes large strains. Hence, we used the material constants determined for human brain at high rates and large strains, but we scaled these constants to match the long-term shear modulus of the rat brain at the middle cortex region. The CSF and ventricles were modelled with a one-term Ogden model with μ = 20 kPa, α = 2 and 50MPa bulk modulus (Ghajari *et al.*, 2017). The dura was modelled with a 31.5 MPa elastic modulus and 0.45 Poisson's ratio (Mao *et al.*, 2006).

Tissue	Density	μ_1 [Pa]	α_1	μ_2 [Pa]	α_2	Bulk modulus
Brain	1040	29.5	10.1	-66.0	-12.9	50
τ_i [ms]	τ_1 = 0.001	τ_2 = 0.01	τ_3 = 0.1	τ_4 = 1	τ_5 = 10	τ_6 = 100
G_i [kPa]	G_1 = 175.5	G_2 = 42.8	G_3 = 3.4	G_4 = 4.4	G_5 = 0.05	G_6 = 1.6

Supplementary Table 1: Material properties of brain tissue

2 Animal experiments

2.1 Animals and husbandry

Male Sprague Dawley rats, acquired from Charles River (Margate, United Kingdom, at approximately 8-9 weeks of age, targeting a weight of ~ 300g at time of surgery), were housed in pairs in individually ventilated cages on autoclaved bedding (wood-shavings) under enrichment conditions (cardboard tunnel, spatulas and paper shredding). The facility provided a 12 hours light/dark cycle with controlled temperature and humidity. Animal had *ad-libitum* access to standard rodent chow (Rat and Mouse No.1 Maintenance, Special diets service, UK) and sterile water. All procedures were carried out in the light phase, except for some welfare checks. Rats were weighed and handled daily for 5-10 minutes prior to procedures for at least a week by the main experimenter (CKD). Animals we supplied with jelly (Hartley's strawberry, 1:2 jelly/water) during that time. After baseline MRI, animals were housed with previous cage-mates after 3-4 hours and subjected to surgery with a minimal resting period of 48 hours. After surgery, buprenorphine was added to the jelly and fed (0.3 mg/kg, based on their current body weight) to the single-housed animals as analgesic.

2.2 MRI acquisition

All animals were transferred to the imaging facilities 24 hours prior to scan and housed with the same cage-mates and identical conditions as before. Anaesthesia was induced with 5% isoflurane (in ~1.5 L/min O_2) and maintained at 2-2.5%. Rats were positioned in the imaging system using ear bars to minimize movement. Body temperature (at 37°C using a warm water heating system) and breathing rate (50-70 breaths/min) were monitored and maintained throughout the experiment.

Invasive surgery	Isoflurane anaesthesia<br>(5% induction, 2-2.5% maintenance, ~1.5 L/min O_2)<br>Buprenorphine analgesia (0.05 mg/kg s.c.)
Procedure for craniotomy	Microdrill, 0.45 mm drill bit
Craniotomy size	rectangular, ~6 mm
Impactor angle/measurement	23°, stereotaxic arm

Impactor tip/shape	5 mm flat, bevelled edge, Leica Impact One standard
Impactor rigidity	steel, not measured
Impactor depth setting	1/2 mm (mild/moderate), manually through stereotaxic arm
Impactor velocity	Set to 3.95-4.05, confirmed with high-speed videography
Surface material	Rigid, head fixed in stereotaxic frame

Supplementary Table 2: Common data elements for CCI injury

2.3 Surgery

Anaesthesia was induced with 5% isoflurane (in ~1.5 L/min O_2) and maintained with 2-2.5%. Buprenorphine (0.05 mg/kg s.c.; Vetergesic, UK) in sterile saline was administered as a perioperative analgesic at least 30 min before first incision. The scalp area was shaved, disinfected, and anaesthetized animals transferred to a stereotaxic frame. Body temperature was adjusted and maintained at 37°C using a rectal probe and a heating pad.

All following surgical procedures were performed under aseptic conditions. A midline incision was performed using additional local anaesthesia (Lidocaine, 1%; Mercury Pharma, Ireland) and the subcutaneous tissue and periosteum carefully retracted. A ~6 mm unilateral rectangular craniotomy, -0.5mm to -6.5 mm posterior and + 3.5 mm lateral to Bregma, was performed on each animal, using a high-speed microdrill with a 0.45 mm drill bit. Any bleeding was controlled through topical application of adrenaline-soaked (0.075 mg/kg; Hameln, UK) sterile gelatine sponges (Surgispon, Aegis Pharmaceuticals, India). The drill area was constantly irrigated with sterile saline and bone dust removed with a surgical suction unit. The bone flap was stored in sterile saline for later reimplantation.

Injury was induced with a 5 mm flat electromagnetically driven steel impactor (23° angle), using the Leica Impact One (Leica Microsystems, UK) by positioning the impactor perpendicular to the surface of the dura mater. The impactor struck the exposed dura at ~4 m/s for 100 milliseconds with an impact depth of either 1 or 2 mm, set through the stereotaxic arm. Common data elements for CCI are described in Supplementary Table 2 (Smith *et al.*, 2015). Based on previous classification and our MRI and histology data, injury would be classified as mild (1 mm) or moderate (2 mm) (Siebold *et al.*, 2018). Following impact, the dura was briefly covered with saline-soaked gelatine sponges to control potential epidural bleeding and inspected for signs of rupture. In one animal, this was found, and the craniotomy was only covered with absorbable gelatine sponges. In all other animals, the bone flap was re-implanted and the craniotomy was sealed with a nontoxic light-curing resin (Technovit 2200; Kulzer, Germany). After irrigation and cleaning, the subcutaneous tissue and scalp were sutured with

Vicryl Plus (5-0 and 4-0; Ethicon, UK). Animals received warmed saline (s.c.) and were allowed to recover under additional oxygen (10 min) and gentle warming. Following recovery, all animals were single-housed and checked for signs of pain and discomfort every 30 minutes for the following 4 hours. From the next day on, all animals were weighted and inspected twice daily for the first 72 hours and afterwards every morning. Buprenorphine was given every 12 hours (0.3 mg/kg, p.o.) for at least 5 days, based on a scoring system (Morton and Griffiths, 1985). Following this period, rats were housed in the same pairs as before, with cage-mates subjected to the same type of impact.

Fourteen days post-impact, animals were subjected to a second MRI. On the following day, rats were subjected to deep pentobarbital (Euthanal, Boehringer Ingelheim, UK) anaesthesia, followed by transcardial perfusion with ice-cold PBS containing heparin (10 units/mL) and 4% PFA in PBS. Brains were postfixed for 48 hours in 4% PFA, equilibrated in 30% sucrose with 0.05% NaN_3 and stored in PBS (with 0.05% NaN_3) at 4°C.

2.4 Randomization and blinding

Baseline acquisition of MRI (n=18) was performed in animals not yet assigned to any group (by MYL, NB, CKD). On the day of surgery, cages containing two animals were randomly assigned to either mild or moderate impact (n=10, n=11, respectively), sham-surgery (3) or designated as drug- and surgery-naïve controls (4) by a single investigator (CKD). Post-injury MRI acquisition was performed unblinded (MYL, NB, CKD). Analysis of MRI datasets was performed blinded (RS), however under supervision (MYL). Immunohistochemistry, histopathology and quantitative analysis were also performed by blinded investigators (FM, PD), again under supervision (CKD). Final statistical analysis was performed unblinded. It should be noted that reliable blinding in experimental animal models of TBI is difficult to achieve, as injured animals are identifiable due to inherent pathology of the injury in all employed outcome measures.

3 MRI

3.1 T1 and T2 MRI analysis

Baseline-timepoint images:

Supplementary Figure 1 shows an overview of both the baseline and post-injury processing of the MRI data. T2 baseline images were registered to their T1 counterparts with FSL FLIRT affine-only registration, limiting the angle rotations to 10° or less. A group template was then created using T1 and T2 images in T1 space with ANTs

(antsMultivariateTemplateConstruction.sh script). The publicly available rat atlas (Waxholm Space Atlas), containing T2/DWI data, brain and GM/WM masks, was registered to the T2 group template (ANTs, non-linear registration).

Post-injury-timepoint images:

T2 images were registered to their T1 counterparts similarly to described before. Brain extraction was performed in T1 individual space using antsBrainExtraction.sh, with the previously created T2 group template and mask as inputs. Semi-automatic segmentation, using IMSEG v1.8, was conducted to delineate brain areas with focal lesions in the T2 individual space images. Finally, an affine-only registration was performed on the resulting skull-stripped T2 images to the group template space using FSL FLIRT, with the lesion masks supplied as argument to the -inweight parameter. This step was necessary to remove the confounding effect of the hyper-intense lesions in the linear affine registration. Morphological differences in the boundary of the cortex with the skull in the injured animals were controlled by limiting the angle rotations in all dimensions to 10° or less and masking the individual brains.

Lesion segmentation:

Semi-automatic segmentation, using IMSEG v1.8, was conducted to delineate brain areas with focal lesions. T2 images were imported into the software and lesion maps were drawn as overlays. To generate the lesion probability distribution, binary lesion masks were transformed to group space using ANTS, followed by concatenation of masks to display the regions of increasing lesion burden using FSL (see Figure 3).

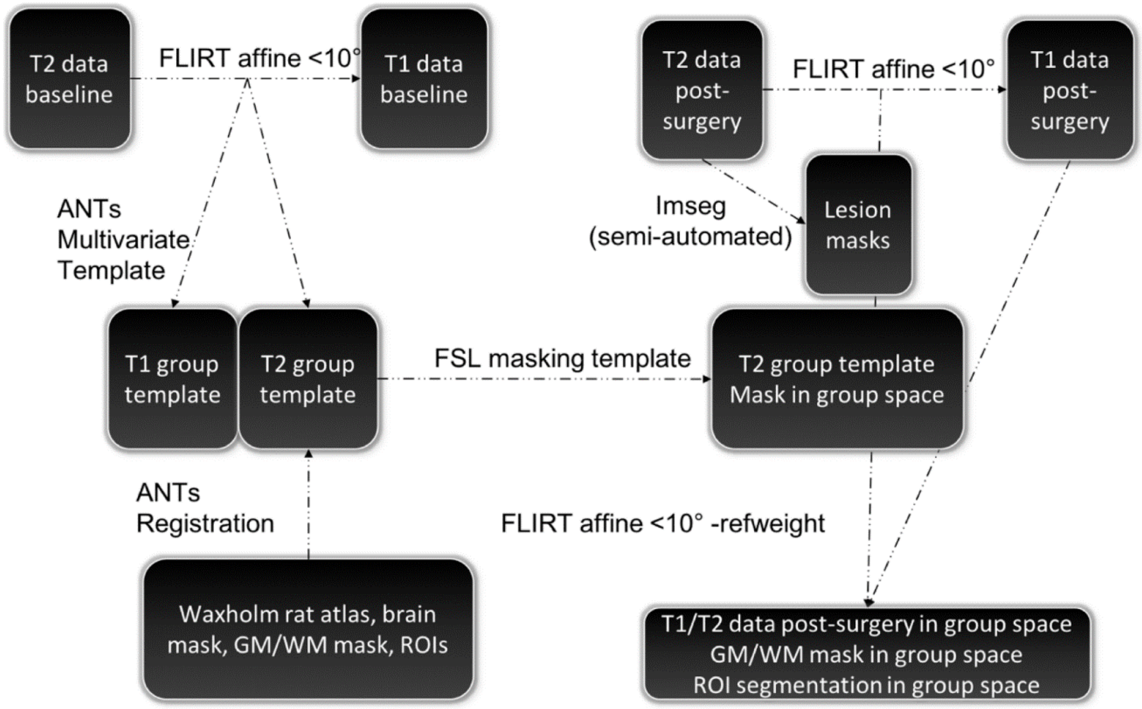

Supplementary Figure 1: MRI registration pipeline

Registration pipeline, from individual space to group space, incorporating the segmentation and masks from the Waxholm rat atlas. Software used includes FSL for linear registration, ANTs for template creation and registration, and IMSEG for semi-automated lesion drawing.

3.2 DTI analysis of the whole CC

Additional to investigating DTI measures in the individual segments of the corpus callosum (CC), we performed an analysis of the whole CC.

For fractional anisotropy, impact resulted in a decrease following moderate impact. A two-way ANOVA (with timepoint and hemisphere as factors) revealed a significant main effect of timepoint (baseline vs. d14) [F (2, 72) = 14.57, P<0.0001], hemisphere (ipsi- or contralateral) [F (1, 72) = 5.344, P=0.0237] and a significant interaction [F (2, 72) = 5.102, P=0.0085] (Supplementary Figure 2A). Tukey's post hoc test indicated significantly reduced FA in the ipsilateral CC after moderate impact (d14) compared to d0 baseline [q(72.00)=6.647, P=0.002], mild impact (d14) [q(72.00)=7.135, P<0.0001] and contralateral CC after moderate impact (d14) [q(72.00)= 4.918, P=0.0107].

Mean diffusivity was found to be changed after impact (Supplementary Figure 2B). Two-way ANOVA showed significant main effect of timepoint [F (2, 72) = 23.24, P<0.0001], but not of hemisphere or any interaction.

Orientation dispersion values in the whole CC were found to be increased following impact (Supplementary Figure 2 C). Two-way ANOVA showed a significant main effect of timepoint [F (2, 72) = 24.92, P<0.0001] and hemisphere [F (1, 72) = 16.06, P=0.0001], again with a significant interaction [F (2, 72) = 9.318, P=0.0003]. Tukey's post-hoc test showed that OD in the ipsilateral CC was significantly increased in animals subjected to moderate impact (d14) when compared to baseline (d0) [q(72.00)= 11.14, P<0.0001], mild impact (d14) [q(72.00)= 7.983, P<0.0001] and contralateral CC after moderate impact (d14) [q(72.00)= 7.674, P<0.0001].

Neurite density was found to be increased after impact (Supplementary Figure 2D). A two-way ANOVA revealed a significant main effect for timepoint [F (2, 72) = 29.84, P<0.0001], with a trend for hemisphere [F (1, 72) = 3.470, P=0.0666], but no interaction.

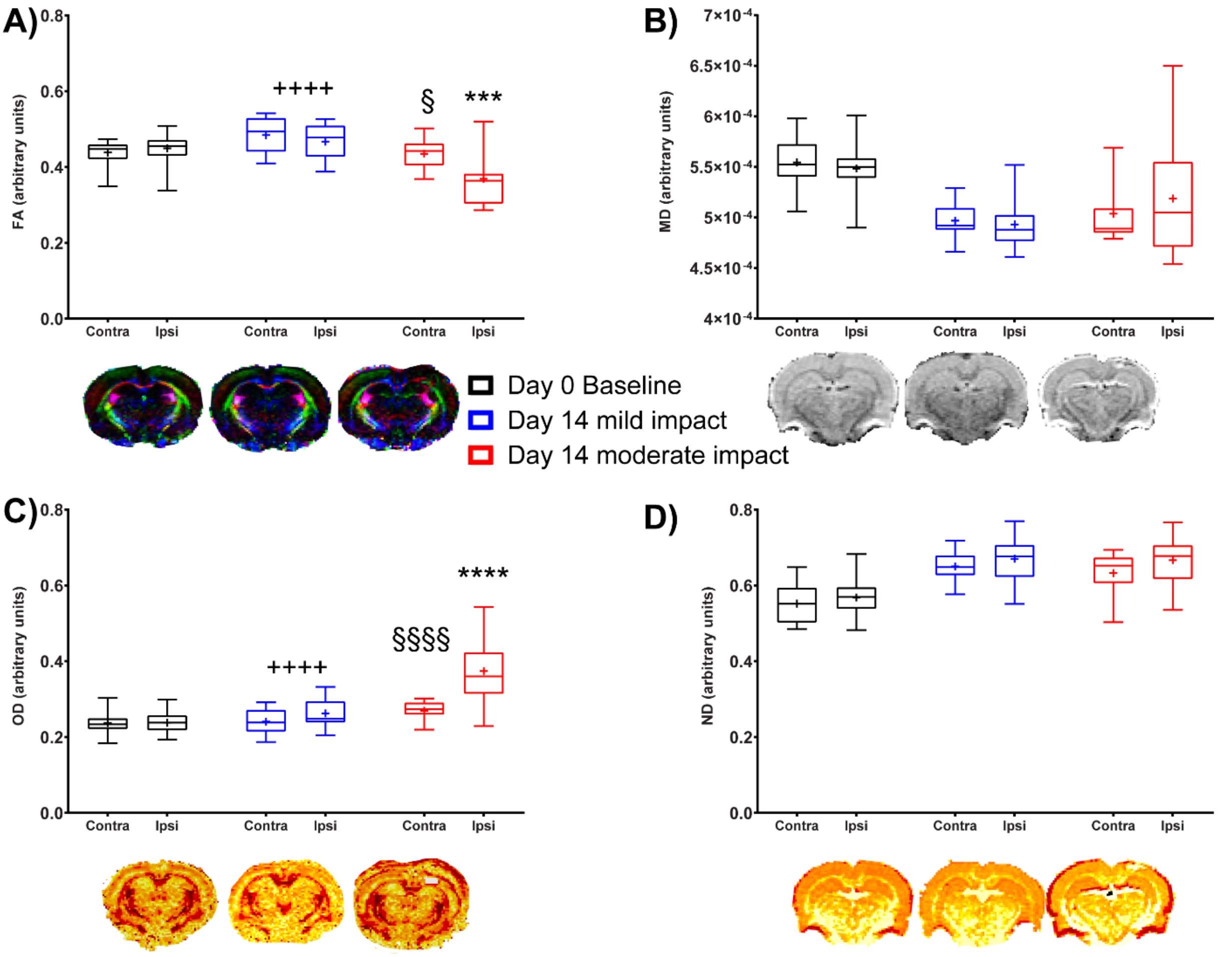

Supplementary figure 2: Diffusion MRI measures in the whole corpus callosum

Diffusion tensor and neurite orientation dispersion and density-imaging values for the whole corpus callosum at baseline or following mild or moderate impact.

Mean values of A) fractional anisotropy (FA); B) mean diffusivity (MD); C) orientation dispersion (OD); neurite density (ND). Representative maps from each group are shown below the graphs.

* P<0.05, ** P<0.01, *** P<0.001 and **** P<0.0001 as compared to baseline d0 ipsilateral

+ P<0.05, ++ P<0.01, +++ P<0.001 and ++++ P<0.0001 as compared to moderate impact ipsilateral CC d14

§ P<0.05, §§ P<0.01, §§§ P<0.001 and §§§§ P<0.0001 as compared to moderate impact, day 14 ipsilateral to contralateral side

Box-and-Whisker plots, with interquartile interval, minimum, maximum and + indicating the mean. Baseline: n=18, mild impact: n=10, moderate impact: n=11.

4 Histopathology

4.1 Tissue processing and staining

PFA-fixed brains of animal subjected to MRI were blocked with a 3D-printed brain matrix derived from the averaged post-injury scans (Figure 1D). Block 4 was paraffin embedded and serial coronal sections (7 µm) were cut on microtome (Shandon Finesse E+, ThermoFisher Scientific, UK). In total, 3 sections were mounted and analysed per slide (Super Frost Plus, Thermo Fisher Scientific, UK), with every 5th collected, thereby roughly covering 100 µm.

4.1.1 Luxol fast blue staining of the Corpus callosum

For Luxol Fast Blue staining, paraffin sections were deparaffinised and rehydrated via decreasing ethanol concentrations. Slides were then incubated in the LFB (RAL Diagnostics, France) solution overnight at 60°C. Slides were then washed for 5 s in ethanol and destained in a saturated lithium carbonate solution for ~10 s, followed by deionized and tap water wash. Slides were then stained for 5 min in 1% Periodic Acid, followed by a wash in tap water for 5-10 min and incubation in Schiffs Reagent (VWR, UK) for 10 min, followed by another wash in water for 10 min. Slides were counterstained with Mayer's Haematoxylin (TCS Biosciences, UK) for 5 min, dehydrated in increasing concentrations of ethanol and xylene and coverslipped.

4.1.2 Immunohistochemistry for microglia and astrocytes

For immunostaining for microglia (IBA1) and astrocytes (GFAP), paraffin sections were deparaffinized and rehydrated. Afterwards, sections were permeabilised and endogenous peroxidase activity quenched in 1% H_2O_2 in 1xPBS + 0.3% triton-X (w/v) for 30 min, followed by antigen retrieval in 0.01M citrate buffer (pH 6) for 20 min in a steam bath. Sections were then incubated with the primary antibodies, IBA1 (polyclonal, Rabbit IgG, #019-19741, Wako, Japan, 1:15,000 of 0.5 mg/mL) and GFAP (polyclonal, Rabbit IgG, #Z0334, DAKO, Denmark, 1:10,000 of 2.9 mg/mL) in 1x PBS + 0.3% Triton-X at 4°C overnight. On the next day, the Super Sensitive Polymer-HRP IHC detection system (BioGenex, USA) was used following manufacturers protocol. Slides were then stained with DAB for 35 to 90 s under microscope control, depending on the antibody and counterstained with Mayer's Haematoxylin for 5 min, dehydrated in increasing concentrations of ethanol and xylene and coverslipped.

4.1.3 Immunofluorescence staining for neurofilaments

For the immunofluorescent visualization of neurofilaments, paraffin sections were deparaffinized and rehydrated. Afterwards, sections were subjected to antigen retrieval in 0.01M citrate buffer (pH 6) for 20 min in a steam bath. Slides were then blocked for 60 min

[10% (v/v) donkey serum (Vector Labs, USA), 1% BSA (w/v) in PBS with 0.1% (w/v) Triton-X) and incubated with the primary antibody NF (monoclonal, Mouse IgG, M0762, DAKO, Denmark, 1:200) in diluent (2% goat serum, 0.2% BSA, 0.02%Triton-X in 1xPBS) overnight at 4°C. On the next day, sections were incubated with the secondary fluorescent antibody (Goat anti-Mouse IgG, Alexa Fluor 568, Invitrogen UK, #A-11004; 1:200 of 2 mg/mL, in 2% goat serum, 0.2% BSA, 0.02%Triton-X in 1xPBS) for 1 hour at 4°C. Slides were then quenched with a Sudan Black solution, and coverslipped with Vectashield (containing DAPI, Vector Labs, USA).

4.1.4 Image acquisition and histology quantification

Following staining, full sections were imaged using an automated slide scanner (Zeiss Axioscan Z1, Zeiss, Germany) with a 20x objective (Plan Apochromat 20x/0.8), yielding a scaling of 0.22 µm x 0.22 µm per pixel. For immunofluorescence, half sections containing the entirety of the Corpus callosum were imaged on a motorized fluorescent microscope (Zeiss Axio Observer Z1) with a 10x objective (Fluar 10x/0.50), yielding a scaling of 1.3 µm x 1.3 µm per pixel.

CC thickness analysis was performed in FIJI and the CC was divided into five equally sized segments from the midline to the extent of the hippocampus. Thickness of the CC was measured every 500 µm using the line tool, beginning from the midline. Two measurements per segment were averaged to yield the mean thickness per segment. These values were normalized to the contralateral segment of the same section and the final value expressed as % change of contralateral, with 100% indicating no difference between the two hemispheres.

Advanced quantitative analysis for IBA1 and GFAP positive cells was performed using HALO® microglia module. The distance from the midline (center of the dorsal 3rd ventricle) to the extent of the CC at the auditory cortex was measured using a straight line. The measured distance was divided by five and rectangular boxes of the exact size were distributed across the CC (Figure 1D, histology analysis) to guide the placement of five regions of interest (ROI) over the ipsilateral CC. Within these boxes, the area containing the white matter was outlined. Size and location were cross-checked using LFB staining and MRI images. On the contralateral side, the same approach was used. Tissue folds and holes were excluded using the HALO exclusion tool. Then, HALOs microglia module was used to detect IBA1 and GFAP positive cells and their morphological parameters. The parameters of the microglia module were modified based on the detection in the three groups. Number of positive cells and cells classified as “activated” (process thickness >2.7 µm) were normalized to the ROI area to yield the number of IBA1/GFAP positive cells/mm^2 (cell density). Average process length and area of microglia

are reported in µm. Neurofilament staining was quantified using the HALO® Area quantification for fluorescence. CC segments were defined similar as for the IBA1/GFAP quantification and the average fluorescence intensity for Alexa 568 was measured in these segments. This measure determines the intensity for each pixel, which is then normalized for the number of pixels. To account for potential intensity differences across sections and animals, each ipsilateral segment was normalized to the contralateral segment of the same section and the final value expressed as % change of contralateral, with 100% indicating no difference between the two hemispheres.

4.2 Morphological parameters of IBA+ cells in the corpus callosum indicate activated microglia

Morphological parameters of IBA1 positive cells in the CC

In the ipsilateral hemisphere, ANOVA indicated a main effect of impact for both average process area [F(2, 80) = 9.027, P=0.0003] and process length [F(2, 80) = 5.243, P=0.0073], without any effect of segment or interaction. However, post-hoc comparison only indicated significantly lower process length in segment 5 of moderately injured when compared to naïve/sham animals. In the contralateral hemisphere, no changes of average process area or length were noted.

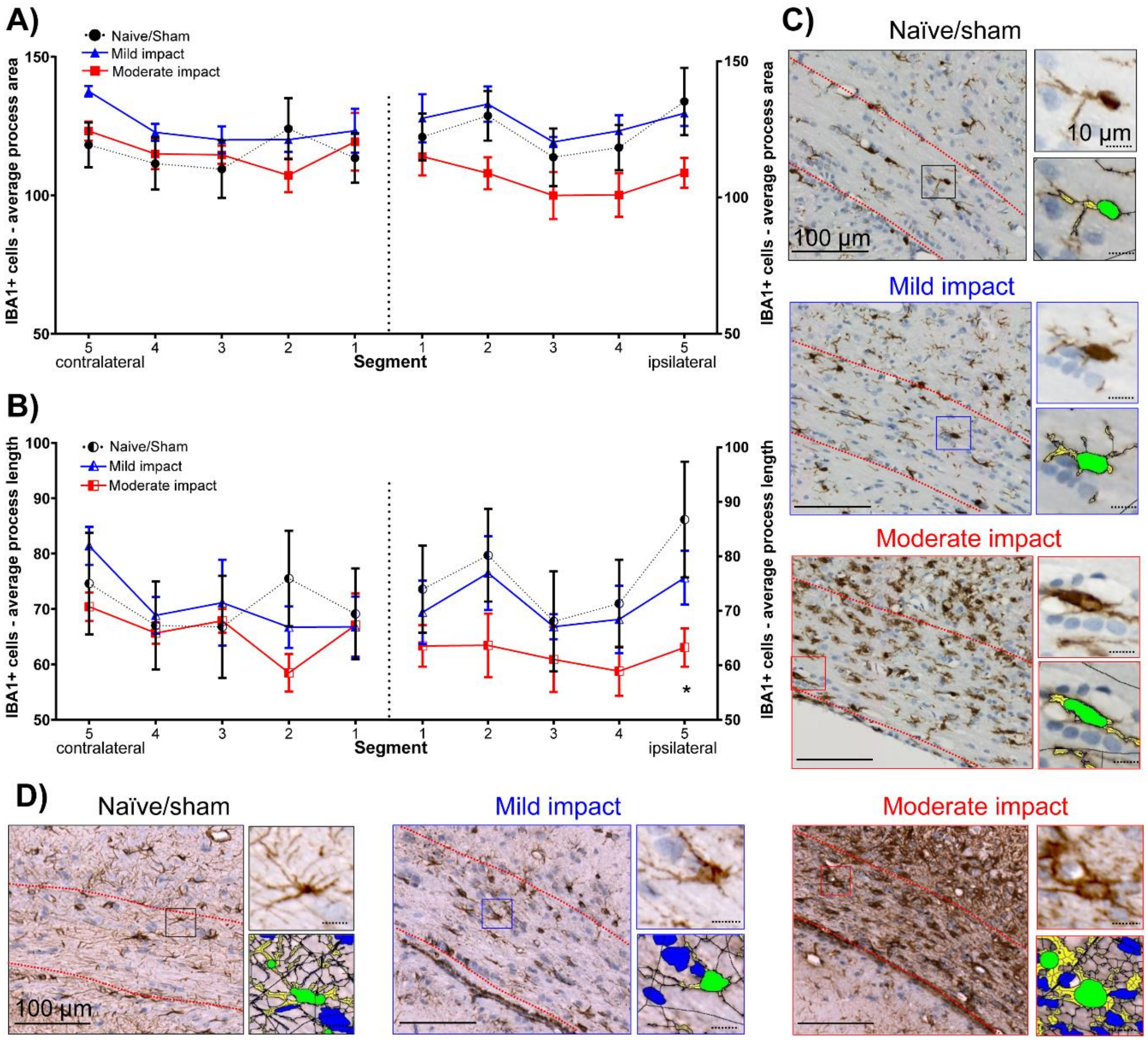

Supplementary Figure 3: Process parameters of IBA1+ cells in the corpus callosum

Quantification of process parameters of IBA1+ cells across segments of the ipsilateral and contralateral corpus callosum (dotted outline).

A) Average process area in µm of IBA1+ cells.

B) Average process length in µm of IBA1+ cells

C) IBA1+ cells exemplifying morphological changes of process length and process area for Naïve/sham (upper panel), mild impact (middle panel) and moderate impact (lower panel). HALO® overlay shows the cell body in green and processes in yellow.

D) GFAP+ cell exemplifying morphological changes of process length and process area for Naïve/sham (left panel), mild impact (middle panel) and moderate impact (right panel). HALO® overlay shows the cell body in green and processes in yellow.

Solid scale bars correspond to 100 µm and dotted bars to 10 µm.

All data is mean±standard error of the mean.

Naive/sham: n=7; mild impact: n=6, moderate impact: n=6.

*, **, ***, **** indicate significant difference ($P<0.05$, 0.01, 0.001 and 0.0001) of moderate impact vs. Naive/sham animals.

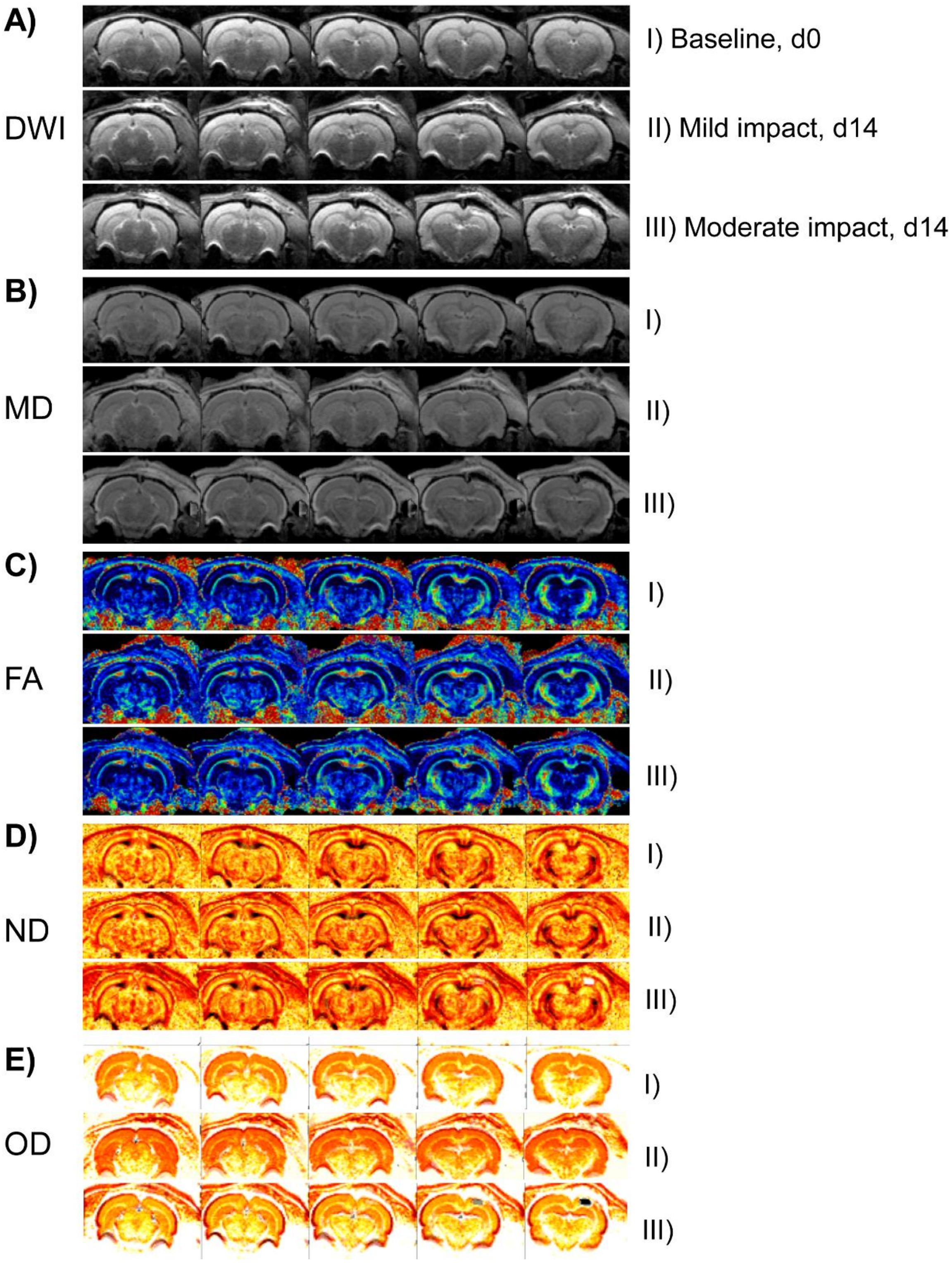

Supplementary Figure 4: Raw MRI data

Raw images of and around the analysed Corpus callosum segments under baseline conditions (all rows designated I, d0) and following mild (all rows designated II) and moderate impact (all rows designated III) at 14 days post injury. Raw data shows:

A) Diffusion weighted data (DWI), following FSL pre-processing,

B/C) Diffusion Tensor Imaging metrics, fractional anisotropy and mean diffusivity (FA, MD). obtained from DTIFIT (FSL).

D/E) Neurite Orientation Dispersion and Density Imaging metrics, neurite density and orientation dispersion (ND, OD), from AMICO NODDI model fitting.

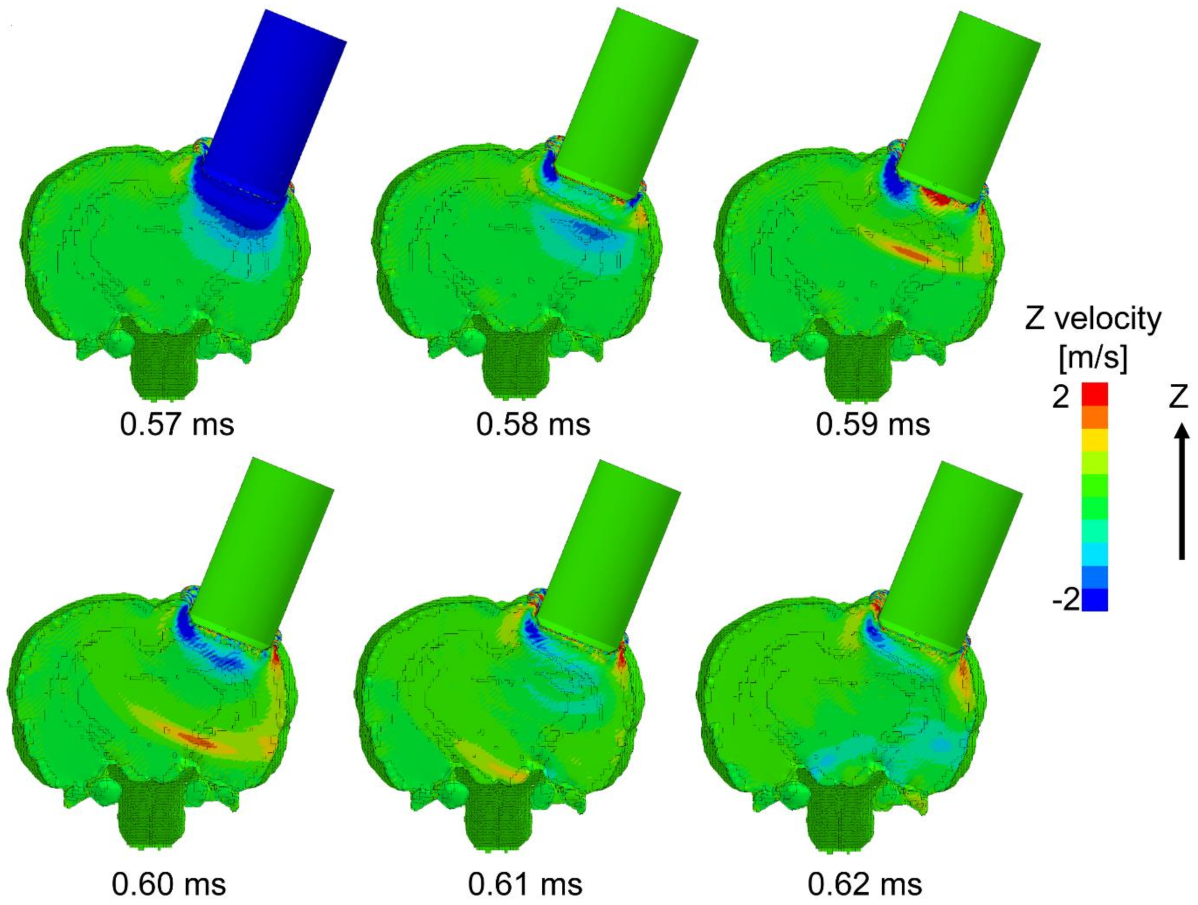

Supplementary Figure 5: Contour plot of the z velocity field predicted for moderate impacts.

The plot shows that during the indentation, the tissue in the vicinity of the impactor has a negative z-velocity (0.57ms). When the impactor stops at the end of the 2mm indentation (0.58ms), a band of positive z-velocity expands into the tissue and propagates towards the opposite side (times 0.59, 0.60 and 0.61 ms). This local increase in velocity is the reason for the local increase in the strain rate in the tissue (Figure 2F, 0.6ms).

References

Finan JD, Elkin BS, Pearson EM, Kalbian IL, Morrison B. Viscoelastic properties of the rat brain in the sagittal plane: effects of anatomical structure and age. Annals of biomedical engineering 2012; 40(1): 70-8.

Ghajari M, Hellyer PJ, Sharp DJ. Computational modelling of traumatic brain injury predicts the location of chronic traumatic encephalopathy pathology. Brain : a journal of neurology 2017; 140(2): 333-43.

Mao H, Zhang L, Yang KH, King AI. Application of a finite element model of the brain to study traumatic brain injury mechanisms in the rat. Stapp car crash journal 2006; 50: 583-600.

Morton DB, Griffiths PH. Guidelines on the recognition of pain, distress and discomfort in experimental animals and an hypothesis for assessment. Vet Rec 1985; 116(16): 431-6.

Siebold L, Obenaus A, Goyal R. Criteria to define mild, moderate, and severe traumatic brain injury in the mouse controlled cortical impact model. Exp Neurol 2018; 310: 48-57.

Smith DH, Hicks RR, Johnson VE, Bergstrom DA, Cummings DM, Noble LJ, *et al*. Pre-Clinical Traumatic Brain Injury Common Data Elements: Toward a Common Language Across Laboratories. J Neurotrauma 2015; 32(22): 1725-35.
