# Supplementary figures and images for: From biomechanics to pathology: predicting axonal injury from patterns of strain after traumatic brain injury
Source: Brain. 2021 Jan 17;144(1):70–91. doi: 10.1093/brain/awaa336 (PMC7990483; doi:10.1093/brain/awaa336)

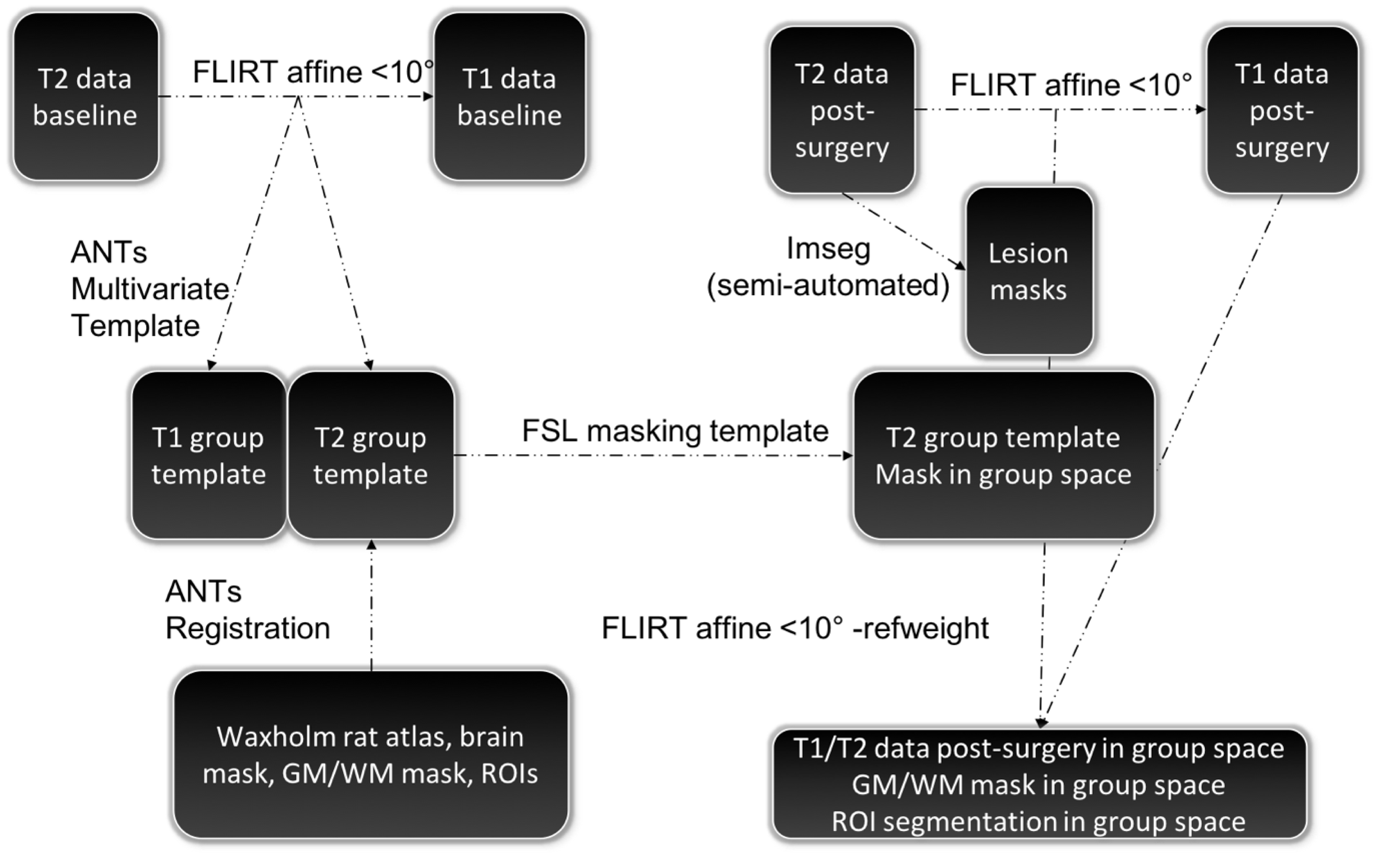

Supplement: awaa336_Supplementary_Data [file awaa336_supplementary_data.zip › OP-BRAI200339_AuthorCorr_AttachmentsFolder_Suppl_Figure 1_MRI_pipeline[AU].tif]
